# COVID-19 pandemic and Farr’s law: A global comparison and prediction of outbreak acceleration and deceleration rates

**DOI:** 10.1371/journal.pone.0239175

**Published:** 2020-09-17

**Authors:** Kevin Pacheco-Barrios, Alejandra Cardenas-Rojas, Stefano Giannoni-Luza, Felipe Fregni

**Affiliations:** 1 Spaulding Research Institute, Spaulding Rehabilitation Hospital and Massachusetts General Hospital, Harvard Medical School, Boston, Massachusetts, United States of America; 2 Unidad de Investigación para la Generación y Síntesis de Evidencias en Salud, Universidad San Ignacio de Loyola, Lima, Peru; 3 Department of Epidemiology, Harvard T.H. Chan School of Public Health, Boston, Massachusetts, United States of America; University of Central Florida, UNITED STATES

## Abstract

The COVID-19 outbreak has forced most of the global population to lock-down and has put in check the health services all over the world. Current predictive models are complex, region-dependent, and might not be generalized to other countries. However, a 150-year old epidemics law promulgated by William Farr might be useful as a simple arithmetical model (percent increase [R1] and acceleration [R2] of new cases and deaths) to provide a first sight of the epidemic behavior and to detect regions with high predicted dynamics. Thus, this study tested Farr’s Law assumptions by modeling COVID-19 data of new cases and deaths. COVID-19 data until April 10, 2020, was extracted from available countries, including income, urban index, and population characteristics. Farr’s law first (R_1_) and second ratio (R_2_) were calculated. We constructed epidemic curves and predictive models for the available countries and performed ecological correlation analysis between R_1_ and R_2_ with demographic data. We extracted data from 210 countries, and it was possible to estimate the ratios of 170 of them. Around 42·94% of the countries were in an initial acceleration phase, while 23·5% already crossed the peak. We predicted a reduction close to zero with wide confidence intervals for 56 countries until June 10 (high-income countries from Asia and Oceania, with strict political actions). There was a significant association between high R_1_ of deaths and high urban index. Farr’s law seems to be a useful model to give an overview of COVID-19 pandemic dynamics. The countries with high dynamics are from Africa and Latin America. Thus, this is a call to urgently prioritize actions in those countries to intensify surveillance, to re-allocate resources, and to build healthcare capacities based on multi-nation collaboration to limit onward transmission and to reduce the future impact on these regions in an eventual second wave.

## Introduction

Covid-19 has been a global public health crisis for different reasons. This pandemic has had a rapid global spread, and in five months, 210 countries were affected by hundreds or thousands of cases and deaths [1]. Also, there was a lack of proper preparation, and it truly stressed the health care system, especially in countries where the incidence was higher, for instance, in China, Iran, and Italy [1].

One of the challenges in this crisis was the lack of good prediction models. In fact, a recent review of 27 studies analyzing 31 different models concluded that models have overall poor predictability and indeed should not be used to drive clinical care decisions [2]. There were several complex models limited to specific geographical regions that give us a partial perspective of this pandemic. It could also lead us to overestimate the number of cases and deaths when we try to replicate the models in other regions [3–6]. One can say that this would be theoretically beneficial as it would overprepare a health system for a rapid surge in cases, and thus, if the numbers are proven to be smaller, that would not have a large detrimental effect [7].

Another challenge in the COVID-19 epidemics is to design models for a condition with limited data. However, a similar scenario may repeat with another unknown or less studied agent. There have been simple prediction models that could have been best used to understand the numbers and dynamics of COVID-19 pandemic easily but from a planetary standpoint due to its accessible computation. In this article, we will discuss a simple and elegant method to forecast epidemic dynamics proposed almost 200 years ago by William Farr.

**Fig 1:**
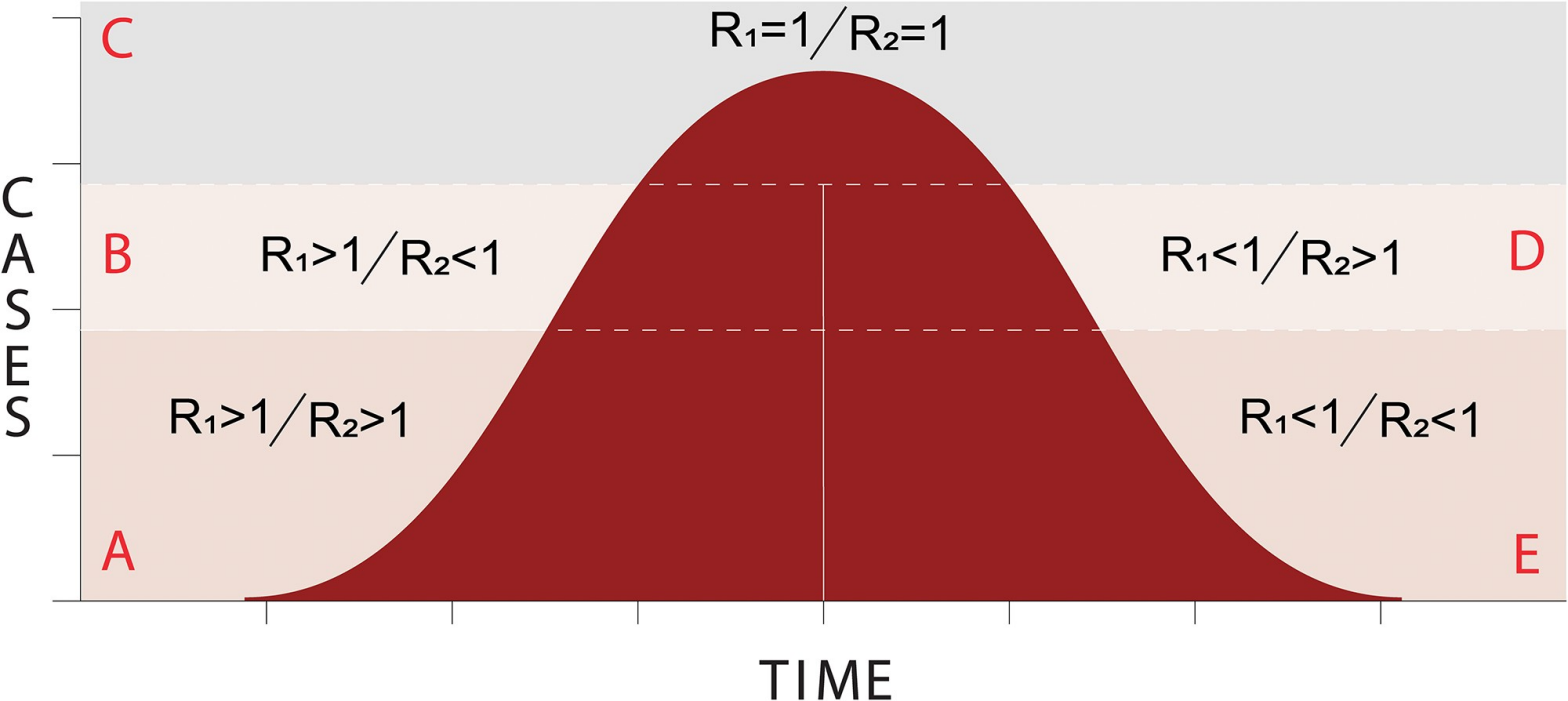
Theoretical framework of epidemic phases based on Farr’s law.

Farr’s law stated that the epidemic’s dynamic could be described as the relation of two arithmetic ratios. The first ratio (R_1_) represents the change of cases or deaths comparing one time against the immediately before time. The second ratio (R_2_) measures the rate of change of R_1_, or in mathematical terms, the acceleration of the estimate (new cases or deaths) [8, 9]. Using this concepts and assumptions, we proposed a theoretical framework to classify the epidemic phase (Fig 1) in a specific region and time as following: i) Phase A—both high change of events and acceleration; ii) Phase B—high change of events but low acceleration; iii) Phase C—no change of events nor acceleration; iv) Phase D—small change of events but higher acceleration; and v) Phase E—both small change of events and acceleration.

Farr’s law has not been widely used as other models using differential-equations were instead employed, such as the susceptible-infectious-removed (SIR) model [10]. The main reason is that it does not consider other important variables as population characteristics (immunity and susceptibility), public health interventions, and political actions against the pandemic. However, it may still be valuable and especially for new outbreaks where there is a lack of knowledge on parameters of disease such as Ebola [11], Chikungunya [11], opioid abuse [12], and indeed the current COVID-19 pandemic. In the study of 1990, Bregman et al. [9] showed a good prediction model for the cases of AIDS, showing that the peak was close, and it would happen a rapid deceleration which it did take place in the following years. Santillan et al. [11] compared the Farr’s model with the incidence decay with exponential adjustment (IDEA) and SIR models. They reported Farr’s Law mathematical approach to resemble solutions of SIR model in an asymptotic limit, where changes of transmission respond both to control policies and depletion of susceptible individuals. Moreover, they suggested the concept of the reproduction number (R_0_) and the effects of control of epidemics (via behavior change or public health policy intervention) were implicitly captured in Farr’s work (pre-microbial era).

In this study, we will model COVID-19 current data (until April 10, 2020) of new confirmed cases and deaths, from 210 countries as to test the assumptions of the 1840 Farr’s law, to describe the epidemic dynamics, and also to make predictions to identify areas with high dynamic and suggest preparation and actions of health system in those regions.

## Methods

### Data

We extracted the COVID-19 data of total and new confirmed cases and deaths from all countries available in “Worldometer” website (210 countries, see Table 1) [13], until April 10, 2020. This website provides a real-time update on the actual number of COVID-19 cases worldwide, including the total confirmed cases, daily new confirmed cases, and severity of the disease (recovered, critical condition, or confirmed death) by country. Worldometer is composed of a team of researchers, developers, and volunteers with no political, governmental, or corporate affiliation to provide time relevant world statistics. As general information, it has been voted as one of the best free reference websites by the American Library Association, and its data has been used in the United Nations Conference Rio+20 [14].

**Table 1:** Total cases, deaths, and Farr’s ratios associated with COVID-19 pandemic, per country until April 10, 2020.

							Cases										Mortality			
Region	Country	Income	Urban population (%)	Population Density Pop/Km^2^	Adults older than 65 y	Any Restriction Policy	First case reported*	Total Cases on April 10	Cases/Population per 100,000 inhab·	First ratio	Percent increase	Second ratio	Epidemic Phase	Predicted End Date	Epidemic Status June 10	New Cases on June 10	Total Deaths on April 10	First ratio	Percent increase	Second ratio
North America	Canada	High	81·41	4·08	17·23	Yes	21-Feb	22,046	58·41	1·47	46·67%	0·71	B	25-May	Descending	472	556	2·71	171·49%	1·46
	Greenland	High	86·82	0·14	··	··	18-Mar	11	19·38	0·42	-58·33%	0·17	E	15-Abr	No cases*	0	··	··	··	··
	Mexico	Upper Middle	80·16	64·91	7·22	Yes	29-Mar	3,441	2·67	1·68	68·39%	0·84	B	> Jun-10	Increasing	4,199	194	3·20	219·84%	1·03
	USA	High	82·26	35·77	15·81	Yes	21- Feb	489,268	147·81	1·52	51·63%	0·70	B	30-May	Descending	20,852	18,015	2·73	172·59%	0·78
Central America	Belize	Upper Middle	45·72	16·79	4·74	··	25-Mar	10	2·51	2·38	137·50%	2·09	A	··	··	··	2	··	··	··
	Costa Rica	Upper Middle	79·34	97·91	9·55	··	8-Mar	539	10·58	0·97	-2·94%	0·88	E	25-May	Increasing (2nd peak)	86	3	0·00	-100·00%	··
	El Salvador	Lower Middle	72·02	309·88	8·29	··	21-Mar	117	1·80	1·65	64·71%	1·04	A	··	··	··	6	0·75	-25·00%	0·00
	Guatemala	Upper Middle	51·05	160·95	4·81	··	16-Mar	126	0·70	1·54	54·22%	1·25	A	··	··	··	3	0·00	-100·00%	··
	Honduras	Lower Middle	57·10	85·69	4·69	Yes	14-Mar	382	3·86	1·45	45·15%	0·70	B	15-May	Increasing	485	23	1·33	33·00%	0·06
	Nicaragua	Lower Middle	58·52	53·73	5·25	··	20-Mar	7	0·11	0·83	-16·67%	1·08	D	·	··	··	1	··	··	··
	Panama	High	67·71	56·19	8·10	Yes	11-Mar	2,752	63·78	1·43	42·90%	0·75	B	20-May	Increasing	656	66	1·95	94·62%	0·82
South America	Argentina	Upper Middle	91·87	16·26	11·12	Yes	5-Mar	1,894	4·19	1·40	40·43%	0·65	B	15-May	Increasing	1,226	81	2·37	136·62%	0·99
	Bolivia	Lower Middle	69·43	10·48	7·19	Yes	12-Mar	268	2·30	2·56	156·16%	2·10	A	··	··	··	19	0·86	-14·29%	0·49
	Brazil	Upper Middle	86·57	25·06	8·92	Yes	29-Feb	18,397	8·65	1·65	65·24%	0·85	B	> Jun-10	Plateau peak	33,100	974	2·23	122·77%	0·80
	Chile	High	87·56	25·19	11·53	Yes	4-Mar	6,501	34·01	1·66	65·92%	0·80	B	5-Jun	Increasing	5,737	65	2·28	127·53%	0·77
	Colombia	Upper Middle	80·78	44·75	8·48	Yes	9-Mar	2,223	4·37	1·47	47·48%	1·09	A	··	··	··	69	2·26	125·61%	1·00
	Ecuador	Upper Middle	63·82	68·79	7·16	Yes	1-Mar	7,161	40·59	1·66	65·66%	1·82	A	··	··	··	297	1·69	68·98%	0·72
	Falkland Islands	··	··	0·25	··	··	5-Apr	5	143·68	··	··	··	··	··	··	··	··	··	··	··
	French Guiana	··	··	3·39	··	··	11-Mar	83	27·79	1·69	68·57%	1·81	A	··	··	··	·	0·00	-100·00%	··
	Guyana	Upper Middle	26·61	3·96	6·45	··	12-Mar	37	4·70	··	··	··	··	··	··	··	6	0·67	-33·33%	0·00
	Paraguay	Upper Middle	61·59	17·51	6·43	··	10-Mar	129	1·81	1·84	83·94%	1·51	A	··	··	··	6	0·00	-100·00%	0·00
	Peru	Upper Middle	77·91	24·99	8·09	Yes	7-Mar	5,897	17·88	2·76	175·64%	1·77	A	··	··	··	169	2·54	154·29%	0·83
	Suriname	Upper Middle	66·06	3·69	6·91	··	20-Mar	10	1·70	··	··	··	··	··	··	··	1	··	··	··
	Uruguay	High	95·33	19·71	14·81	··	14-Mar	473	13·62	0·91	-9·30%	0·87	E	20-May	Descending	1	7	3·00	200·00%	0·00
	Venezuela	Upper Middle	88·21	32·73	7·27	··	15-Mar	171	0·60	0·75	-25·29%	0·84	E	5-May	Descend not clear	106	9	0·83	-16·67%	0·13
Caribbean	Anguilla	··	··	164·84	··	··	2-Apr	3	20·00	··	··	··	··	··	··	··	··	·	··	··
	Antigua and Barbuda	High	24·60	218·83	8·80	··	23-Mar	19	19·40	··	··	··	··	··	··	··	2	·	··	··
	Aruba	High	43·41	588·03	13·55	··	17-Mar	86	80·55	1·32	31·72%	1·18	A	··	··	··	··	··	··	··
	Bahamas	High	83·03	38·53	7·26	··	19-Mar	41	10·43	1·22	22·47%	0·73	B	5-May	Descending	0	8	··	··	··
	Barbados	High	31·15	666·61	15·80	··	19-Mar	66	22·97	0·95	-5·35%	0·89	E	15-May	Descending	4	4	0·25	-75·00%	0·00
	Bermuda	High	100·00	1184·59	··	··	22-Mar	48	77·07	0·80	-20·00%	1·16	D	··	··	··	4	0·00	-100·00%	··
	British Virgin Islands	High	47·72	198·68	··	··	31-Mar	3	9·92	··	··	··	··	··	··	··	··	··	··	··
	Caribbean Netherlands	High	··	··	··	··	2-Apr	2	7·63	··	··	··	··	··	··	··	··	··	··	··
	Cayman Islands	High	100·00	267·39	··	··	19-Mar	45	68·47	2·20	120·33%	1·76	A	··	··	··	1	··	··	··
	Cuba	Upper Middle	77·04	109·00	15·19	Yes	13-Mar	564	4·98	1·84	84·46%	1·00	A	··	··	··	15	1·75	75·00%	1·33
	Curaçao	High	89·15	367·12	16·68	··	14-Mar	14	8·53	0·58	-41·67%	0·93	E	25-Abr	Descending	1	1	··	··	··
	Dominica	Upper Middle	70·48	95·50	··	··	23-Mar	16	22·23	1·50	50·00%	10·25	A	··	··	··	··	··	··	··
	Dominican Republic	Upper Middle	81·07	219·98	7·08	Yes	6-Mar	2,620	24·15	1·37	36·93%	0·54	B	5-May	Plateau peak	393	126	3·50	250·27%	1·93
	Grenada	Upper Middle	36·27	327·81	9·62	··	26-Mar	12	10·66	··	··	··	··	··	··	··	··	··	··	··
	Guadeloupe	··	94·78	245·70	··	··	14-Mar	143	35·74	1·34	34·05%	3·46	A	··	··	··	8	0·44	-55·56%	0·17
	Haiti	Low	55·28	403·60	4·95	··	23-Mar	30	0·26	1·26	26·19%	1·34	A	··	··	··	2	··	··	··
	Jamaica	Upper Middle	55·67	270·99	8·80	··	11-Mar	63	2·13	1·53	53·32%	1·35	A	··	··	··	4	0·25	-75·00%	0·00
	Martinique	··	··	333·33	··	··	10-Mar	154	41·04	0·55	-44·99%	0·60	E	25-Abr	Descending	0	6	0·75	-25·00%	0·00
	Montserrat	··	··	49·02	··	··	26-Mar	9	180·29	··	··	··	··	··	··	··	··	··	··	··
	Saint Kitts and Nevis	High	30·78	201·70	··	··	30-Mar	11	20·68	··	··	··	··	··	··	··	··	0·29	-71·43%	0·88
	Saint Lucia	High	18·68	298·18	9·81	··	15-Mar	14	7·62	0·50	-50·00%	5·00	D	··	··	··	··	··	··	··
	Saint Martin	High	··	698·11	··	··	18-Mar	32	82·76	0·92	-7·78%	0·50	E	25-Abr	No cases*	0	2	··	··	··
	Saint Pierre Miquelon	··	··	24·79	··	··	5-Apr	1	17·26	··	··	··	··	··	··	··	··	··	··	··
	Sint Maarten	High	100·00	1235·29	··	··	23-Mar	50	116·62	3·82	282·46%	2·21	A	··	··	··	8	··	··	··
	St· Barth	High	··	476·19	··	··	16-Mar	6	60·75	··	··	··	··	··	··	··	··	··	··	··
	St· Vincent Grenadines	High	52·20	282·59	9·59	··	1-Apr	12	10·82	··	··	··	··	··	··	··	··	··	··	··
	Trinidad and Tobago	High	53·18	270·93	10·73	··	14-Mar	109	7·79	0·93	-7·22%	2·13	D	··	··	··	8	0·72	-27·78%	0·22
	Turks and Caicos	High	93·10	39·65	··	··	26-Mar	8	20·66	··	··	··	··	··	··	··	1	··	··	··
Africa	Algeria	Upper Middle	72·63	17·73	6·36	··	1-Mar	1,761	4·02	1·25	25·03%	0·96	B	> Jun-10	Descending	102	256	3·10	209·70%	2·00
	Angola	Lower Middle	65·51	24·71	2·22	··	21-mAR	19	0·06	5·63	462·50%	22·06	A	··	··	··	2	··	··	··
	Benin	Low	47·31	101·85	3·25	··	18-Mar	35	0·29	2·03	102·78%	2·07	A	··	··	··	1	··	··	··
	Botswana	Upper Middle	69·45	3·98	4·22	··	31-Mar	13	0·55	··	··	··	··	··	··	··	1	··	··	··
	Burkina Faso	Low	29·36	72·19	2·41	··	10-Mar	443	2·12	1·15	14·51%	0·87	B	30-May	Descending	0	24	5·00	400·00%	1·84
	Burundi	Low	13·03	435·18	2·25	··	2-Apr	3	0·03	··	··	··	··	··	··	··	··	··	··	··
	Cabo Verde	Lower Middle	65·73	134·93	4·61	··	21-Mar	7	1·26	··	··	··	··	··	··	··	1	··	··	··
	Cameroon	Lower Middle	56·37	53·34	2·73	··	14-Mar	803	3·02	2·49	149·24%	1·06	A	··	··	··	12	0·60	··	··
	CAR	Low	41·36	7·49	2·83	··	20-Mar	8	0·17	··	··	··	··	··	··	··	··	··	··	··
	Chad	Low	23·06	12·29	2·48	··	23-Mar	11	0·07	··	··	··	··	··	··	··	··	··	··	··
	Congo	Lower Middle	66·92	15·36	2·68	··	19-Mar	60	1·09	6·64	564·33%	12·19	A	··	··	··	5	0·00	-100·00%	·
	Djibouti	Low	77·78	41·37	4·53	··	23-Mar	150	15·18	2·33	133·21%	1·43	A	··	··	··	1	··	··	··
	DRC	Upper Middle	44·46	37·08	3·02	··	13-Mar	215	0·24	1·37	36·74%	0·98	B	> Jun-10	Descend not clear	131	20	0·73	-26·67%	0·30
	Egypt	Lower Middle	42·70	98·87	5·23	Yes	1-Mar	1,699	1·66	1·59	58·78%	1·18	A	··	··	··	118	1·79	78·64%	0·92
	Equatorial Guinea	Upper Middle	72·14	46·67	2·46	··	18-Mar	18	1·28	0·78	-22·22%	2·22	D	··	··	··	··	··	··	··
	Eritrea	Low	··	29·36	··	··	25-Mar	34	0·96	0·45	-55·21%	0·38	E	··	··	··	··	··	··	··
	Eswatini	Lower Middle	23·80	66·06	4·01	··	22-Mar	12	1·03	··	··	··	··	··	··	··	··	··	··	··
	Ethiopia	Low	20·76	109·22	3·50	··	15-Mar	65	0·06	1·79	78·97%	1·43	A	···	··	··	3	··	··	··
	Gabon	Upper Middle	89·37	8·22	3·56	··	17-Mar	44	1·98	5·38	438·10%	9·62	A	··	··	··	1	··	··	··
	Gambia	Low	61·27	225·31	2·59	··	23-Mar	4	0·17	··	··	··	·	··	··	··	1	··	··	··
	Ghana	Lower Middle	56·06	130·82	3·07	··	15-Mar	378	1·22	2·52	152·34%	1·30	A	··	··	··	6	··	··	··
	Guinea	Low	36·14	50·52	2·93	··	20-Mar	194	1·48	4·61	361·00%	2·93	A	··	··	··	··	··	··	··
	Guinea-Bissau	Low	43·36	66·65	2·82	··	30-Mar	36	1·83	··	··	··	··	··	··	··	··	··	··	··
	Ivory Coast	Lower Middle	50·78	78·83	2·86	··	14-Mar	444	1·68	3·74	273·91%	1·40	A	··	··	··	3	··	··	··
	Kenya	Lower Middle	27·03	90·30	2·34	··	15-Mar	189	0·35	1·47	46·93%	0·64	B	10-May	Descend not clear	105	7	0·50	-50·00%	0·00
	Liberia	Low	51·15	50·03	3·25	··	17-Mar	37	0·73	··	··	··	··	··	··	··	5	0·33	··	0·00
	Libya	Upper Middle	80·10	3·80	4·39	··	28-Mar	24	0·35	··	··	··	··	··	··		1	··	··	··
	Madagascar	Low	37·19	45·14	2·99	··	23-Mar	93	0·34	0·92	-7·94%	1·72	D	··	··	··	··	··	··	··
	Malawi	Low	16·94	192·44	2·65	··	4-Apr	9	0·05	··	··	··	··	··	··	··	1	··	··	··
	Mali	Low	42·36	15·64	2·51	··	26-Mar	87	0·43	1·18	17·62%	1·10	A	··	··	··	7	0·67	-33·33%	0·50
	Mauritania	Lower Middle	53·67	4·27	3·14	··	18-Mar	7	0·15	··	··	··	··	··	··	··	1	··	··	··
	Mauritius	Upper Middle	40·79	623·30	11·47	··	19-Mar	318	25·00	1·05	4·85%	0·77	B	··	··	··	9	1·67	66·67%	2·00
	Mayotte	··	··	695·19	··	··	16-Mar	191	70·01	1·37	37·09%	1·10	A	··	··	··	2		·	·
	Morocco	Lower Middle	62·45	80·73	7·01	··	5-Mar	1,448	3·92	1·88	88·24%	1·09	A	··	··	··	107	4·61	360·86%	2·67
	Mozambique	Low	35·99	37·51	2·89	··	24-Mar	20	0·06	··	··	··	··	··	··	··	··	··	··	··
	Namibia	Upper Middle	50·03	2·97	3·64	··	19-Mar	16	0·63	0·67	-33·33%	1·00	D	··	··	··	··	··	··	··
	Niger	Low	16·43	17·72	2·60	··	22-Mar	410	1·69	4·06	306·18%	1·07	A	··	··	··	11	1·50	50·00%	0·00
	Nigeria	Lower Middle	50·34	215·06	2·75	··	28-Feb	288	0·14	1·42	42·08%	1·23	A	··	··	··	7	1·22	22·22%	1·61
	Réunion	··	99·14	351·65	··	··	12-Mar	382	42·67	0·65	-35·26%	1·23	D	··	··	··	··	··	··	··
	Rwanda	Low	17·21	498·66	2·94	··	15-Mar	113	0·87	0·95	-5·50%	0·77	E	5-May	Increasing	41	··	··	··	··
	Sao Tome and Principe	Lower Middle	72·80	219·82	2·93	··	6-Mar	4	1·83	··	··	··	··	··	··	··	··	··	··	··
	Senegal	Lower Middle	47·19	82·35	3·09	··	3-Mar	265	1·58	1·00	0·50%	0·89	B	25-May	Plateau peak	124	2	0·00	-100·00%	··
	Seychelles	High	56·69	210·35	7·59	··	15-Mar	11	11·18	1·17	16·67%	2·08	A	··	··	··	··	··	··	··
	Sierra Leone	Low	42·06	105·99	2·97	··	1-Apr	8	0·10	··	··	··	··	··	··	··	··	··	··	··
	Somalia	Low	44·97	23·92	2·87	··	26-Mar	21	0·13	1·53	52·50%	1·03	A	··	··	··	1	··	··	··
	South Africa	Upper Middle	66·36	47·63	5·32	··	7-Mar	2,003	3·38	0·99	-0·53%	1·30	D	··	··	··	24	1·75	··	0·49
	South Sudan	Low	19·62	17·71	3·40	··	7-Apr	4	0·04	··	··	··	··	··	··	··	··	··	··	··
	Sudan	Lower Middle	34·64	22·16	3·58	··	18-Mar	15	0·03	2·08	108·33%	1·70	A	··	··	··	2	··	··	··
	Tanzania	Low	33·78	63·58	2·60	··	18-Mar	32	0·05	1·28	27·78%	2·42	A	··	··	··	3	··	··	··
	Togo	Low	41·70	145·05	2·87	··	20-Mar	76	0·92	1·93	92·86%	2·89	A	··	··	··	3	0·00	-100·00%	··
	Tunisia	Lower Middle	68·95	74·44	8·32	··	8-Mar	671	5·68	2·05	105·24%	0·94	B	··	··	··	25	1·53	52·78%	1·27
	Uganda	Low	23·77	213·06	1·94	··	23-Mar	53	0·12	0·20	-80·42%	0·29	E	··	··	··	··	0·13	-86·94%	0·19
	Western Sahara	··	··	2·13	··	··	4-Apr	4	0·67	··	··	··	··	··	··	··	··	··	··	··
	Zambia	Lower Middle	43·52	23·34	2·10	··	22-Mar	40	0·22	··	··	··	··	··	··	··	2	··	··	··
	Zimbabwe	Lower Middle	32·21	37·32	2·94	··	21-Mar	11	0·07	0·83	-16·67%	1·08	D	··	··	··	3	··	··	··
Asia	Afghanistan	Low	25·50	56·94	2·58	··	7-Mar	521	1·34	1·44	43·62%	0·70	B	15-May	Descend not clear	683	15	2·00	100·00%	1·50
	Armenia	Upper Middle	63·15	103·68	11·25	··	12-Mar	937	31·62	1·18	17·79%	0·86	B	30-May	Descend not clear	428	12	0·92	-8·33%	1·69
	Azerbaijan	Upper Middle	55·68	120·27	6·20	··	29-Feb	991	9·77	2·11	111·02%	0·96	B	··	··	··	10	2·17	116·67%	4·13
	Bahrain	High	89·29	2017·27	2·43	··	25-Feb	913	53·66	1·15	14·55%	0·94	B	> Jun-10	Descend not clear	469	6	0·83	-16·67%	0·13
	Bangladesh	Lower Middle	36·63	1239·58	5·16	··	14-Mar	424	0·26	4·34	334·03%	13·66	A	··	··	··	27	1·67	66·67%	8·50
	Bhutan	Lower Middle	40·90	19·78	6·00	··	20-Mar	5	0·65	··	··	··	··	··	··	··	··	··	··	··
	Brunei	High	77·63	81·40	4·87	··	10-Mar	136	31·09	0·37	-63·26%	1·32	D	··	··	··	1	··	··	··
	Cambodia	Lower Middle	23·39	92·06	4·57	··	7-Mar	119	0·71	0·68	-32·18%	1·03	D	··	··	··	··	0·00	-100·00%	··
	China	Upper Middle	59·15	148·35	10·92	··	Before 23-Feb	81,907	5·69	1·02	1·96%	1·21	A	··	··	··	3,336	0·67	-32·79%	0·88
	Cyprus	High	66·81	128·71	13·72	Yes	11-Mar	595	49·28	1·44	43·85%	0·92	B	> Jun-10	Descending	2	10	0·33	-66·67%	0·00
	Georgia	Upper Middle	58·63	65·28	14·87	··	28-Feb	234	5·87	1·57	57·02%	1·08	A	··	··	··	3	··	··	··
	Hong Kong	High	100·00	7096·19	16·88	··	16-Feb	990	13·21	1·01	1·29%	0·85	B	20-May	Descending	0	4	··	··	··
	India	Lower Middle	34·03	454·94	6·18	··	2-Mar	7,598	0·55	2·24	124·42%	1·18	A	··	··	··	246	2·71	170·59%	0·99
	Indonesia	Upper Middle	55·33	147·75	5·86	··	6-Mar	3,512	1·28	1·38	38·10%	0·94	B	> Jun-10	··	··	306	1·33	32·76%	0·89
	Iran	Upper Middle	74·90	50·22	6·18	··	21-Feb	68,192	81·19	1·33	32·54%	1·03	A	··	··	··	4,232	1·01	0·82%	1·02
	Iraq	Upper Middle	70·47	88·53	3·32	··	25-Mar	1,279	3·18	1·48	48·13%	1·02	A	··	··	··	70	1·27	27·41%	0·89
	Israel	High	92·42	410·53	11·98	Yes	23-Feb	10,095	116·63	1·72	71·74%	0·67	B	20-May	Increasing (2nd peak)	175	95	4·80	379·67%	0·38
	Japan	High	91·62	347·07	27·58	Yes	16-Feb	5,530	4·37	1·95	95·03%	1·04	A	··	··	··	99	1·31	30·61%	1·31
	Jordan	Upper Middle	90·98	112·14	3·85	··	15-Mar	372	3·65	1·05	4·68%	1·28	A	··	··	··	7	··	··	··
	Kazakhstan	Upper Middle	57·43	6·77	7·39	··	14-Mar	812	4·32	2·37	137·17%	1·61	A	··	··	··	10	2·00	100·00%	0·69
	Kuwait	High	100·00	232·17	2·55	··	25-Feb	993	23·25	2·24	124·11%	1·43	A	··	··	··	1	··	··	··
	Kyrgyzstan	Lower Middle	36·35	32·93	4·49	··	20-Mar	298	4·57	1·66	65·60%	0·91	B	··	··	··	5	··	··	··
	Laos	Lower Middle	35·00	30·60	4·08	··	25-Mar	16	0·22	1·25	25·00%	3·13	A	··	··	··	··	··	··	··
	Lebanon	Upper Middle	88·59	669·49	7·00	··	26-Feb	609	8·92	0·74	-26·10%	0·83	E	10-May	Descend not clear	20	20	0·79	-20·83%	0·19
	Macao	High	100·00	20777·50	10·48	··	15-Mar	45	6·93	0·51	-48·68%	0·67	E	20-Abr	No cases*	0	··	··	·	·
	Malaysia	Upper Middle	76·04	95·96	6·67	Yes	28-Feb	4,346	13·43	1·14	13·64%	1·03	A	··	··	··	70	0·89	-10·78%	0·59
	Maldives	Upper Middle	39·81	1718·99	3·70	··	8-Mar	19	3·51	··	··	··	··	··	··	··	··	··	··	··
	Mongolia	Lower Middle	68·45	2·04	4·08	··	16-Mar	16	0·49	0·83	-16·67%	1·21	D	··	··	··	··	··	··	··
	Myanmar	Lower Middle	30·58	82·24	5·78	··	24-Mar	27	0·05	0·79	-20·83%	0·84	E	··	··	··	3	··	··	··
	Nepal	Low	19·74	195·94	5·73	··	23-Mar	9	0·03	··	··	··	··	··	··	··	··	··	··	··
	Oman	High	84·54	15·60	2·39	··	25-Mar	484	9·48	1·76	76·48%	1·39	A	··	··	··	3	1·00	0·00%	1·00
	Pakistan	Lower Middle	36·67	275·29	4·31	··	29-Feb	4,695	2·13	1·44	44·27%	1·02	A	··	··	··	66	1·66	65·78%	0·86
	Palestine	Lower Middle	76·16	758·98	··	··	6-Mar	267	5·23	2·11	110·52%	1·18	A	··	··	··	2	·	·	·
	Philippines	Lower Middle	46·91	357·69	5·12	Yes	6-Mar	4,195	3·83	0·92	-7·95%	0·57	E	5-May	Descend not clear	740	221	1·50	50·44%	0·76
	Qatar	High	93·58	239·59	1·37	··	1-Mar	2,512	87·19	2·76	176·45%	2·06	A	··	··	··	6	1·00	0·00%	1·00
	South Korea	High	81·46	529·65	3·31	Yes	16-Feb	10,450	20·38	0·85	-14·52%	0·85	E	20-May	Descending	50	208	0·99	-0·91%	1·02
	Saudi Arabia	High	83·84	15·68	11·46	··	4-Mar	3,651	10·49	1·41	40·65%	0·87	B	> Jun-10	Increasing	3,717	47	3·01	201·28%	0·32
	Singapore	High	100·00	7953·00	14·42	··	16-Feb	2,108	36·03	1·53	52·80%	1·25	A	··	··	··	7	1·00	0·00%	2·13
	Sri Lanka	Upper Middle	18·48	345·56	10·47	··	11-Mar	190	0·89	1·29	29·05%	2·84	A	··	··	··	7	0·72	-27·78%	0·22
	Syria	Low	54·16	92·07	4·50	··	25-Mar	19	0·11	··	··	··	··	··	··	··	2	··	··	··
	Taiwan	High	27·13	655·54	··	··	16-Feb	382	1·60	0·64	-36·32%	0·75	E	30-Abr	Descending	0	6	··	··	··
	Thailand	Upper Middle	49·95	135·90	11·90	··	17-Feb	2,473	3·54	1·40	40·40%	0·70	B	15-May	Descending	4	33	1·97	97·22%	2·11
	Timor-Leste	Lower Middle	30·58	85·27	4·32	··	10-Apr	2	0·15	··	··	··	··	··	··	··	··	··	··	··
	Turkey	Upper Middle	75·14	106·96	8·48	Yes	13-Mar	47,029	55·76	2·18	117·79%	0·90	B	··	··	··	1,006	2·12	112·13%	0·81
	UAE	High	86·52	135·61	1·09	··	16-Mar	3,360	33·97	2·11	110·58%	1·02	A	··	··	··	14	0·33	-66·67%	0·50
	Uzbekistan	Lower Middle	50·48	77·47	4·42	··	16-Mar	624	1·86	2·73	173·19%	2·35	A	··	··	··	3	··	··	··
	Vietnam	Lower Middle	35·92	308·13	7·27	··	6-Mar	257	0·26	0·51	-49·44%	0·95	E	30-Abr	Descending	0	··	1·61	60·52%	0·65
	Yemen	Low	36·64	53·98	2·88	··	10-Apr	1	0·00	··	··	··	··	··	··	··	··	··	··	··
Europe	Albania	Upper Middle	60·32	104·61	13·74	··	9-Mar	416	14·46	0·90	-10·02%	0·87	E	20-May	Increasing (2nd peak)	42	23	6·00	500·00%	0·63
	Andorra	High	88·06	163·84	··	··	15-Mar	601	777·84	1·28	28·27%	1·05	A	··	··	··	26	3·06	205·82%	0·44
	Austria	High	58·30	107·21	19·00	Yes	27-Feb	13,551	150·46	1·13	13·05%	0·63	B	10-May	Descending	26	319	1·83	83·01%	0·77
	Belarus	Upper Middle	78·60	46·73	14·85	··	3-Mar	1,981	20·96	4·35	335·40%	9·17	A	··	··	··	19	2·25	··	0·30
	Belgium	High	98·00	377·21	18·79	Yes	1-Mar	26,667	230·09	1·56	55·86%	0·92	B	··	··	··	3,019	2·30	130·14%	0·65
	Bosnia and Herzegovina	Upper Middle	48·25	64·92	16·47	··	6-Mar	901	27·46	1·34	34·03%	0·75	B	20-May	Increasing (2nd peak)	47	36	2·39	138·89%	0·42
	Bulgaria	Upper Middle	75·01	64·70	21·02	Yes	8-Mar	635	9·14	1·10	10·48%	0·87	B	30-May	Increasing (2nd peak)	104	25	1·71	71·43%	0·15
	Channel Islands	··	30·91	861·11	17·30	··	10-Mar	398	228·92	1·72	71·88%	0·66	B	15-May	Descending	0	9	2·00	100·00%	0·25
	Croatia	Upper Middle	56·95	73·08	20·45	Yes	26-Feb	1,495	36·42	0·97	-2·76%	0·67	E	10-May	Descending	2	21	2·28	127·78%	1·49
	Czechia	High	73·79	137·60	19·42	Yes	2-Mar	5,674	52·98	1·16	15·95%	0·81	B	25-May	Descending	73	119	2·14	114·11%	0·72
	Denmark	High	87·87	138·07	19·81	Yes	28-Feb	5,819	100·46	1·56	55·69%	1·03	A	··	··	··	247	2·42	142·09%	1·14
	Estonia	High	68·88	30·39	19·63	Yes	3-Mar	1,258	94·83	1·63	62·99%	2·20	A	··	··	··	24	5·50	450·00%	0·20
	Faeroe Islands	High	42·06	34·74	··	··	6-Mar	184	376·56	0·70	-30·43%	0·58	E	25-Abr	No cases*	0	··	··	··	··
	Finland	High	85·38	18·16	21·72	Yes	26-Feb	2,769	49·98	1·19	19·49%	0·92	B	> Ju-10	Descending	15	48	4·37	337·41%	1·28
	France	High	80·44	122·34	20·03	Yes	25-Feb	124,869	191·30	1·55	55·44%	0·90	B	··	··	··	13,197	1·88	87·54%	0·83
	Germany	High	77·31	237·37	21·46	Yes	25-Feb	119,624	142·78	1·14	13·75%	0·80	B	30-May	Descending	311^	2,607	2·52	151·72%	0·79
	Gibraltar	High	100·00	3371·80	··	··	16-Mar	127	376·96	3·81	281·11%	6·48	A	··	··	··	··	··	··	··
	Greece	High	79·06	83·22	21·66	Yes	27-Feb	2,011	19·29	1·40	39·90%	1·44	A	··	··	··	91	1·26	26·01%	0·80
	Hungary	High	71·35	107·91	19·16	Yes	5-Mar	1,190	12·32	1·32	31·61%	0·71	B	15-May	Descending	10	77	2·12	112·06%	4·28
	Iceland	High	93·81	3·53	14·80	Yes	1-Mar	1,675	490·85	0·94	-5·76%	0·73	E	10-May	Descending	0	6	1·75	75·24%	1·01
	Ireland	High	63·17	70·45	13·87	Yes	3-Mar	7,054	142·86	1·48	48·38%	0·75	B	25-May	Descending	16	287	0·67	-33·13%	1·15
	Isle of Man	High	52·59	147·50	··	··	20-Mar	201	236·38	1·71	70·54%	1·14	A	··	··	··	1	··	··	··
	Italy	High	70·44	205·45	22·75	Yes	21-Feb	147,577	244·08	0·89	-10·84%	0·87	E	10-Jun	Descending	202	18,849	0·96	-4·21%	0·79
	Latvia	High	68·14	30·98	20·04	Yes	8-Mar	612	32·45	1·16	16·44%	0·67	B	10-May	Descending	3	3	··	··	··
	Liechtenstein	High	14·34	236·94	··	··	11-Mar	79	207·20	1·43	43·04%	6·54	A	··	··	··	1	··	··	··
	Lithuania	High	67·68	44·53	19·71	Yes	28-Feb	999	36·70	1·35	34·99%	0·69	B	15-May	Descending	6	22	3·11	211·11%	4·53
	Luxembourg	High	90·98	250·09	14·18	Yes	5-Mar	3,223	514·87	0·95	-5·16%	0·63	E	5-May	Descending	3	54	5·02	402·38%	32·70
	Malta	High	94·61	1511·03	20·35	··	9-Mar	350	79·27	1·38	37·94%	1·35	A	··	··	··	2	··	··	··
	Moldova	Lower Middle	42·63	123·52	11·47	Yes	10-Mar	1,438	35·65	1·98	97·60%	0·98	B	··	··	··	29	5·85	485·00%	··
	Monaco	High	100·00	19306·93		··	12-Mar	90	229·35	0·97	-3·22%	0·86	E	15-May	Descending	0	1	0·00	-100·00%	··
	Montenegro	Upper Middle	66·81	46·27	14·97	··	18-Mar	255	40·60	1·26	26·31%	1·28	A	··	··	··	2	··	··	··
	Netherlands	High	91·49	511·46	19·20	Yes	28-Feb	23,097	134·80	1·31	31·16%	0·77	B	30-May	Descending	184	2,511	1·66	66·14%	0·70
	North Macedonia	Upper Middle	57·96	82·59	13·67	··	6-Mar	711	34·13	1·26	25·56%	0·81	B	25-May	Descend not clear	125	32	1·71	71·11%	1·20
	Norway	High	82·25	14·55	17·05	Yes	27-Feb	6,298	116·17	1·12	11·84%	0·93	B	> Jun-10	Descending	18	113	1·92	92·38%	1·01
	Poland	High	60·06	124·04	17·52	Yes	6-Mar	5,955	15·73	1·06	6·41%	0·67	B	15-May	Descend not clear	282	181	2·19	118·66%	0·95
	Portugal	High	65·21	112·24	21·95	Yes	3-Mar	15,472	151·74	1·27	26·65%	0·80	B	30-May	Descending	294	435	1·83	83·25%	0·54
	Romania	Upper Middle	54·00	84·64	18·34	Yes	28-Feb	5,467	28·42	2·23	123·46%	1·42	A	··	··	··	270	1·92	92·34%	0·67
	Russia	Upper Middle	74·43	8·82	14·67	Yes	2-Mar	11,917	8·17	2·41	141·05%	0·99	B	··	··	··	94	3·61	260·56%	1·20
	San Marino	High	97·23	563·08	··	··	1-Mar	344	1013·82	1·23	23·38%	1·53	A	··	··	··	34	3·64	264·02%	23·48
	Serbia	Upper Middle	56·09	79·83	18·35	Yes	9-Mar	3,105	35·54	1·97	97·38%	0·91	B	··	··	··	71	2·37	137·30%	0·46
	Slovakia	High	53·73	113·29	15·63	Yes	7-Mar	715	13·10	1·45	44·92%	1·24	A	··	··	··	2	··	··	··
	Slovenia	High	54·54	102·64	19·61	Yes	5-Mar	1,160	55·80	1·06	6·40%	0·96	B	> Jun-10	Descending	2	45	1·75	74·75%	1·13
	Spain	High	80·32	93·53	19·38	Yes	24 -Feb	157,053	335·91	1·22	21·97%	0·73	B	25-May	Descending	314	15,970	1·07	7·21%	0·68
	Sweden	High	87·43	25·00	20·10	Yes	26-Feb	9,685	95·90	1·47	47·28%	0·93	B	·	··	··	870	2·43	142·78%	0·86
	Switzerland	High	73·80	215·52	18·62	Yes	1-Mar	24,551	283·68	1·00	0·12%	0·75	B	20-May	Descending	23	1,001	1·53	53·06%	0·70
	UK	High	83·40	274·83	18·40	Yes	23-Feb	73,758	108·65	1·85	84·70%	0·82	B	> Jun-10	Descending	1,003	8,958	2·48	148·14%	0·91
	Ukraine	Lower Middle	69·35	77·03	16·43	Yes	12-Mar	2,203	5·04	2·18	118·00%	0·81	B	> Jun-10	Plateau peak	525	69	3·09	208·89%	2·41
	Vatican City	High	100	2272·73	··	··	24-Mar	8	998·75	··	··	··	··	··	··	··	··	··	··	··
Oceania	Australia	High	86·01	3·25	15·66	··	20-Feb	6,203	24·33	0·78	-21·83%	0·55	E	5-May	Descending	9	53	4·07	307·41%	12·59
	Fiji	Upper Middle	56·25	48·36	5·45	··	21-Mar	16	1·78	3·00	200·00%	4·00	A	··	··	··	··	··	··	··
	French Polynesia	··	61·83	75·87	8·29	··	13-Mar	51	18·16	1·18	17·94%	1·99	A	··	··	··	··	··	··	··
	New Caledonia	··	70·68	15·54	9·17	··	21-mar	18	6·30	0·30	-70·00%	0·48	E	··	··	··		··	··	··
	New Zealand	High	86·54	18·55	15·65	··	3-Mar	1,283	26·61	3·16	215·66%	0·73	B	5-Jun	No cases*	0	2	··	··	··
	Papua New Guinea	Lower Middle	13·17	19·00	3·45	··	6-Apr	2	0·02	·	··	··	··	··	··	··	··	··	··	··

USA: United States of America; CAR: Central African Republic; DRC: Democratic Republic of the Congo; UAE: United Arab Emirates; UK: United Kingdom; inhab: inhabitants· (··): No data· > June 10: the predicted end date goes beyond that date· *Approximate date of first case reported· A: First and Second ratio higher than 1·0; B: First ratio higher than 1·0 but Second ratio less than 1·0; C: Both ratios are equal to 1; D: First ratio less than 1·0 but Second ratio higher than 1·0; E: both ratios are less than 1·0·

*No cases for more than 10 days··

^Data belong to June 09, on June 10 there was no cases reported··

The countries with end date beyond June 10 are countries having more than zero predicted cases or deaths after we ended our simulation (June 10, 2020), meaning they could continue with the pandemic wave beyond the end our simulation.

In the context of COVID-19 pandemic, it has been a provider for important governmental institutions such as the UK, Thailand, Pakistan, Sri Lanka, and Vietnam Governments as well as for the COVID-19 dashboard by the Center for Systems Science and Engineering (CSSE) at John Hopkins University [15]. Based on its webpage, they obtain the data directly from official reports from the Government’s communication channels or indirectly through local media sources when they are considered reliable.

We downloaded the data on new cases and deaths, separately, per day and country since the first available record (January 23, 2020). We extracted directly from the webpage code source from each plot using a standardized extraction spreadsheet to prevent losing data. Additionally, we extracted data of income, urban index, population density, population size, and proportion of the older population (>65 years old) from the World Bank data repository [16] of the included countries; we estimated the number of cases and deaths per 1000 and 100,000 inhabitants by country, respectively. Finally, we performed a post-hoc extraction of the new cases for June 10, 2020 (one point extraction), and the political actions against COVID-19 per country, from the University of Washington Health Index and Evaluation Center (IHME) database [17]. We used this analysis to compare current data with our predictions on the COVID-19 worldwide trend, including the countries with reduced cases near zero, and the countries with high predicted dynamics. We exported the data to a standardized spreadsheet in Excel Microsoft 2019 to performed data cleaning.

### Calculation of ratios

According to Farr’s law, the intrinsic epidemic’s behavior could be described as the relation of two arithmetic ratios:

1. The first ratio (R1) represents the change of cases or deaths (first level dynamic) comparing one time against the immediately before time (could be in days or months, based on the natural history of the disease). Thus, by subtracting 1·0 from this ratio, we can calculate the percent increase of cases or deaths, in physical terms, this measure could be understood as the “velocity of spread” of the epidemic.
2. The second ratio (R2) measures the rate of change of R1s. It compares the R1 of one time against the R1 from the immediately before time. In physical terms, we can interpret this ratio as the acceleration of the epidemic (new cases or deaths).

We calculated the Farr’s ratios based on prior reports [9, 11, 12], the first ratio (R_1_) was the division of given cases or deaths (I) at *t* time over that estimate of *t* time before (formula 1). Then, dividing the first ratio of one time over the immediately before resulted in the second ratio (R_2_)(formula 2).

$$R_{1}\;=\;\left(\frac{I(t+1)}{I(t)}\right)$$ (1)

$$R_{2}\;=\;\frac{\left(\frac{I(t+3)}{I(t+2)}\right)}{\left(\frac{I(t+1)}{I(t)}\right)}$$ (2)

We predefined the sum of events over five days of consecutive data as a time length to calculate the ratios, as was suggested in prior studies to use clustered data to stabilize the data distribution [11]. We constructed the epidemic curves of all the 210 countries using those clustered data on new cases and deaths. The normality of the data was examined using skewness and kurtosis without data transformations to assure the estimates’ interpretability. The countries with disease data less than 15 days (to calculate at least one R_2_) were excluded from the ratio’s calculation analysis.

### Prediction models and statistical analysis

We fit a normal curve to these data to use Farr’s law as a predictive model of epidemic dynamics. First, similar to previous studies [9, 11, 12], we assumed the future R_1_ and R_2_ values would be the same as the mean of the past three-time intervals (five days), then we predicted the future first ratio and daily confirmed new cases and deaths, for the next two months (until June 10, 2020), by back-calculation, using the mean of the last three calculated R_2_ [9]. To fit a normal curve, we set two assumptions [9, 11]: i) the R_2_ was constant, having a value between 0·0 and 1·0, which signifies a constant deceleration in the rate of change (R_1_). ii) each included country should report data of three-time intervals at minimum. The countries which do not fulfill these criteria were excluded from the predictive analysis [11].

Additionally, we performed ecological correlation analyses to assess the relationship between R_1_ and R_2_ of new cases and deaths, with urban index, population density, cases/1000 inhabitants, and deaths/100,000 inhabitants. The predefined hypothesis was higher R_1_, and R_2_ ratios are correlated with higher numbers of cases [9, 11] adjusted by population size, and these ratios are related to the number of urban areas and population density in the country.

We calculated 95% confidence intervals using the standard error of the mean of the last three calculated R_2_. Two-sided p<0·05 was considered statistically significant. The analyses were performed in Microsoft Excel 2019 and Stata 15 (StataCorp LLC: College Station, TX).

For the report of this study, we followed the Guidelines for Accurate and Transparent Health Estimates Reporting (GATHER) to define best reporting practices for studies that compute health estimates for multiple populations in different times and spaces [18]. The checklist is reported in S1 Table. This study is exempt from institutional review board’s review due to the use of publicly available and de-identified information.

## Results

### Worldwide COVID-19 dynamics based on R_1_ and R_2_

We obtained information from 210 countries, including reported new confirmed cases and deaths until April 10, as well as their approximate first report date of Covid-19 (Table 1). From them, it was possible to calculate the R_1_ and R_2_ of Covid-19 cases and deaths of 170 countries; it was not possible to estimate neither of the ratios in 40 countries due to limited data available for these countries. The R_1_ and R_2_ ranged from 0·20 to 6·64 and from 0·17 to 22·06, respectively. Our calculation showed that 73 countries (42·94%) are in the epidemic phase A (initial accelerated phase), 57 (33·52%) were decelerating getting closer to the peak (phase B), and 40 (23·5%) were already on the other side of the epidemic curve (phase D and E). For deaths, the R_1_ was calculated for 116 countries with a range between 0 and 6, while for the R_2_ only in 96 (range, 0 to 32·7). The majority of them, 74 (66·07%), showed an increase in mortality ratios (R_1_ and R_2_). Due to absent data on mortality, 75 and 112 countries lacked R_1_ and R_2_ calculation, respectively. As an overall, the world is in A and B epidemic phases and is increasing death rates (45·8%) (Table 1).

**Fig 2:**
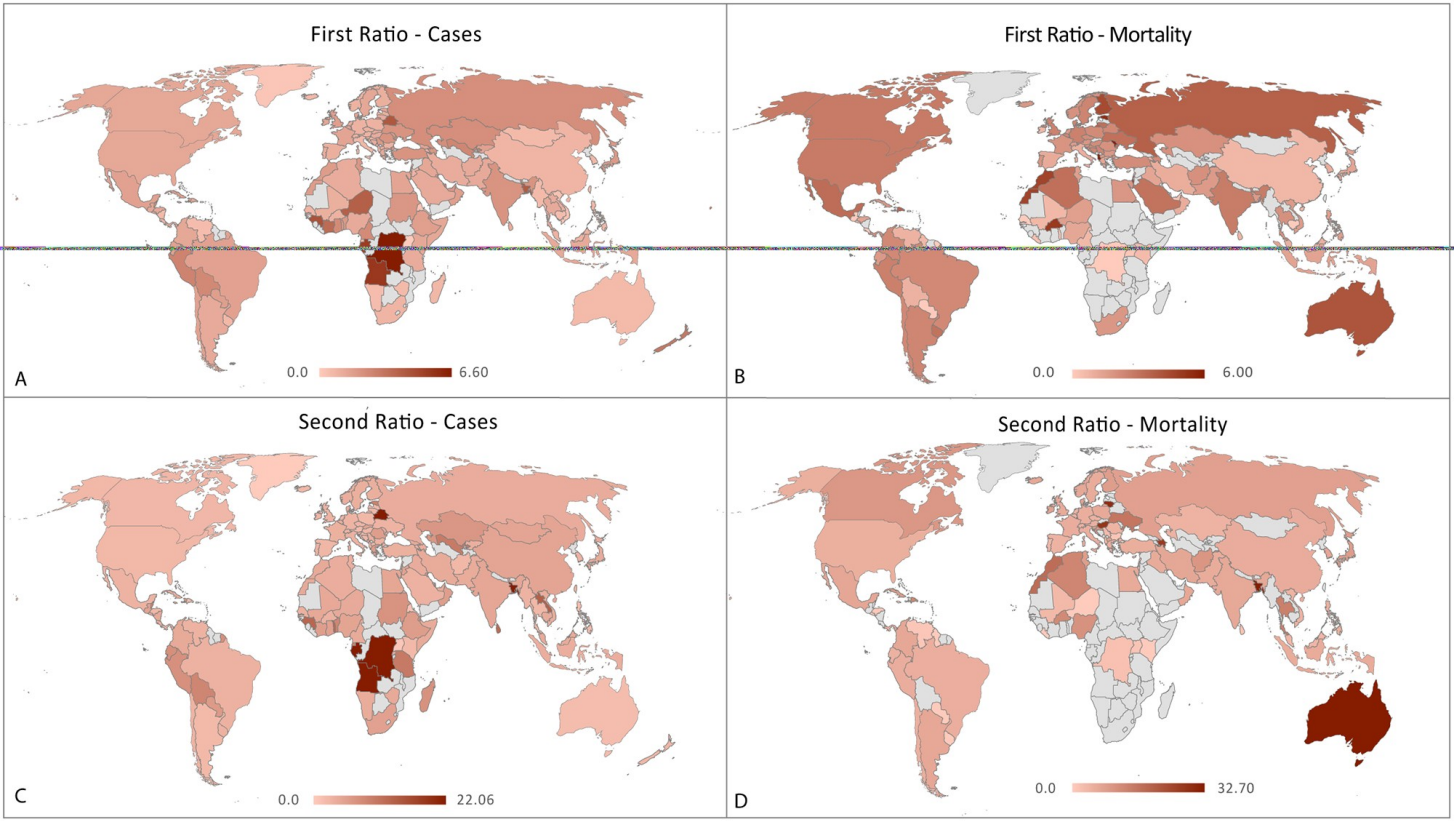
Geographical representation of Farr’s ratios of the COVID-19 pandemic. First ratios represent a percent increase in the epidemic dynamic, and second ratios represent epidemic acceleration. A and B depict the first ratios for the new cases and deaths, respectively. C and D represent the second ratios for new cases and deaths, respectively. The calculations are based on worldwide data until April 10, 2020.

From the 20 countries with highest R_1_ (range, 2·38 to 6·64) and R_2_ (range 2·20 to 22·06) for Covid-19 new cases the majority were from Africa (around 40%, Fig 2) and had a middle-income economy (70% for R_1_ and 35% for R_2_). In case of deaths the range of R_2_ were from 2·73 to 6 and for R_2_ from 1·33 to 32·70. The predominant region for both ratios was Europe, while high income countries were predominant for the R_1_ and middle-income for R_2_ (Table 2). In general terms, from all the countries with high R_1_ low income countries represented 9·92% (13 countries) and 3·75% (3 countries) for cases and deaths respectively, whereas for high R_2_ 12·79% (11 countries) for cases and 5·71% (2 countries) for deaths.

**Table 2:** Countries with higher Farr’s ratios associated with COVID-19 pandemic.

Order	Country	Cases							Order	Country		Deaths			
		Income	Population Density (inhab/km^2^)	Adults ≥65y (%)	Total	First Ratio	Epidemic status on June 10	New Cases on June 10			Income	Population Density (inhab/km^2^)	Adults ≥65y (%)	Total	First Ratio
1	Congo	Lower Middle	15·36	2.68	60	6·64	Descend not clear	45^	1	Albania	Upper Middle	104·61	13.74	23	6·00
2	Angola	Lower Middle	24·71	2.22	19	5·63	Increasing	17	2	Moldova	Lower Middle	123·52	11.47	29	5·85
3	Gabon	Upper Middle	8·22	3.56	44	5·38	Descend not clear	47	3	Estonia	High	30·39	19.63	24	5·50
4	Guinea	Low	50·52	2.93	194	4·61	Descend not clear	42^	4	Luxembourg	High	250·09	14.18	54	5·02
5	Belarus	Upper Middle	46·73	14.85	1,981	4·35	Plateau	801	5	Burkina Faso	Low	72·19	2.41	24	5·00
6	Bangladesh	Lower Middle	1239·58	5.16	424	4·34	Increasing	3190	6	Israel	High	410·53	11.98	95	4·80
7	Niger	Low	17·72	2.6	410	4·06	Descending	0	7	Morocco	Lower Middle	80·73	7.01	107	4·61
8	Sint Maarten	High	1235·29	-	50	3·82	No cases*	0	8	Finland	High	18·16	21.72	48	4·37
9	Gibraltar	High	3371·80	-	127	3·81	Descending	0	9	Australia	High	3·25	15.66	53	4·07
10	Ivory Coast	Lower Middle	78·83	2.86	444	3·74	Increasing	186	10	San Marino	High	563·08	-	34	3·64
11	New Zealand	High	18·55	15.65	1,283	3·16	No cases*	0	11	Russia	Upper Middle	8·82	14.67	94	3·61
12	Fiji	Upper Middle	48·36	5.45	16	3·00	No cases*	0	12	Dominican Republic	Upper Middle	219·98	7.08	126	3·50
13	Qatar	High	239·59	1.37	2,512	2·76	Descend not clear	1716	13	Mexico	Upper Middle	64·91	7.22	194	3·20
14	Peru	Upper Middle	24·99	8.09	5,897	2·76	Descend not clear	5087	14	Lithuania	High	44·53	19.71	22	3·11
15	Uzbekistan	Lower Middle	77·47	4.42	624	2·73	Increasing (2nd peak)	103	15	Algeria	Upper Middle	17·73	6.36	256	3·10
16	Bolivia	Lower Middle	10·48	7.19	268	2·56	Increasing	695	16	Ukraine	Lower Middle	77·03	16.43	69	3·09
17	Ghana	Lower Middle	130·82	3.07	378	2·52	Descend not clear	291^	17	Andorra	High	163·84	-	26	3·06
18	Cameroon	Lower Middle	53·34	2.73	803	2·49	Descend not clear	369	18	Saudi Arabia	High	15·68	3.31	47	3·01
19	Russia	Upper Middle	8·82	14.67	11,917	2·41	Plateau	8404	19	Uruguay	High	19·71	14.81	7	3·00
20	Belize	Upper Middle	16·79	4.74	10	2·38	Descending	0	20	USA	High	35·77	15.81	18,015	2·73
**Order**	**Country**	**Income**	**Population Density (inhab/km**^**2**^**)**		**Total**	**Second Ratio**	**Epidemic status on June 10**	**New Cases on June 10**	**Order**	**Country**	**Income**	**Population Density (inhab/km**^**2**^**)**		**Total**	**Second Ratio**
1	Angola	Lower Middle	24·71	2.22	19	22·06	Increasing	17	1	Luxembourg	High	250·09	14.18	54	32·70
2	Bangladesh	Lower Middle	1239·58	5.16	424	13·66	Increasing	3190	2	San Marino	High	563·08	-	34	23·48
3	Congo	Lower Middle	15·36	2.68	60	12·19	Descend not clear	45^	3	Australia	High	3·25	15.66	53	12·59
4	Dominica	Low	95·50	-	16	10·25	Descending	0	4	Bangladesh	Lower Middle	1239·58	5.16	27	8·50
5	Gabon	Upper Middle	8·22	3.56	44	9·62	Descend not clear	47	5	Lithuania	High	44·53	19.71	22	4·53
6	Belarus	Upper Middle	46·73	14.85	1,981	9·17	Plateau	801	6	Hungary	High	107·91	19.16	77	4·28
7	Liechtenstein	High	236·94	-	79	6·54	No cases*	0	7	Azerbaijan	Upper Middle	120·27	6.20	10	4·13
8	Gibraltar	High	3371·80	-	127	6·48	Descending	0	8	Morocco	Lower Middle	80·73	7.01	107	2·67
9	Saint Lucia	High	298·18	9.81	14	5·00	Descending	0	9	Ukraine	Lower Middle	77·03	16.43	69	2·41
10	Fiji	Upper Middle	48·36	5.45	16	4·00	No cases*	0	10	Singapore	High	7953·00	11.46	7	2·13
11	Guadeloupe	·	245·70	-	143	3·46	Descending	0	11	Thailand	Upper Middle	135·90	11.90	33	2·11
12	Laos	Lower Middle	30·60	4.08	16	3·13	No cases*	0	12	Mauritius	Upper Middle	623·30	3.14	9	2·00
13	Guinea	Low	50·52	2.93	194	2·93	Descending	42^	13	Algeria	Upper Middle	17·73	6.36	256	2·00
14	Togo	Low	145·05	2.87	76	2·89	Descend not clear	21	14	Dominican Republic	Upper Middle	219·98	7.08	126	1·93
15	Sri Lanka	Upper Middle	345·56	10.47	190	2·84	Descending	10	15	Burkina Faso	Low	72·19	2.41	24	1·84
16	Tanzania	Low	63·58	2.6	32	2·42	No cases*	0	16	Armenia	Upper Middle	103·68	11.25	12	1·69
17	Uzbekistan	Lower Middle	77·47	4.42	624	2·35	Increasing (2nd peak)	103	17	Nigeria	Lower Middle	215·06	2.75	7	1·61
18	Equatorial Guinea	Upper Middle	46·67	2.46	18	2·22	No cases*	0	18	Afghanistan	Low	56·94	2.58	15	1·50
19	Sint Maarten	High	1235·29	-	50	2·21	No cases*	0	19	Croatia	Upper Middle	73·08	20.45	21	1·49
20	Estonia	High	30·39	19.63	1,258	2·20	Descending	11	20	Canada	High	4·08	17.23	556	1·46

inhab: inhabitants; USA: United States of America.

*No cases in more than 10 days.

^Cases from June 09, no cases were reported on June 10.

We then calculated the median R_1_ and R_2_ rates for new cases according to the epidemic phase (Fig 1). While R_1_ median rate starts with 1·69 (for phase A) and then decreases to 0·79 in phase E; R_2_ median rate starts with 1·42 for phase A and decreases to 0·81 in phase B but then increases again in phase D to 1·23 to then decrease in phase E to 0·75 thus being aligned with Farr’s law.

#### Correlational analyses

Regarding the correlation analysis, the R_1_ for mortality was positively correlated with urban index (rho = 0·2, p = 0·03), and with deaths per 100 000 inhabitants (rho = 0·3, p = 0·001). No significant correlations were found for the rest of the ratios.

#### Predictive analyses

For the prediction of new cases, we included 69 countries out of 210 countries, the rest of them did not meet the assumption criteria or have enough data. On the other hand, for the prediction of mortality, 64 countries were included in the modeling (in S2 Table and in S3 Table).

**Fig 3:**
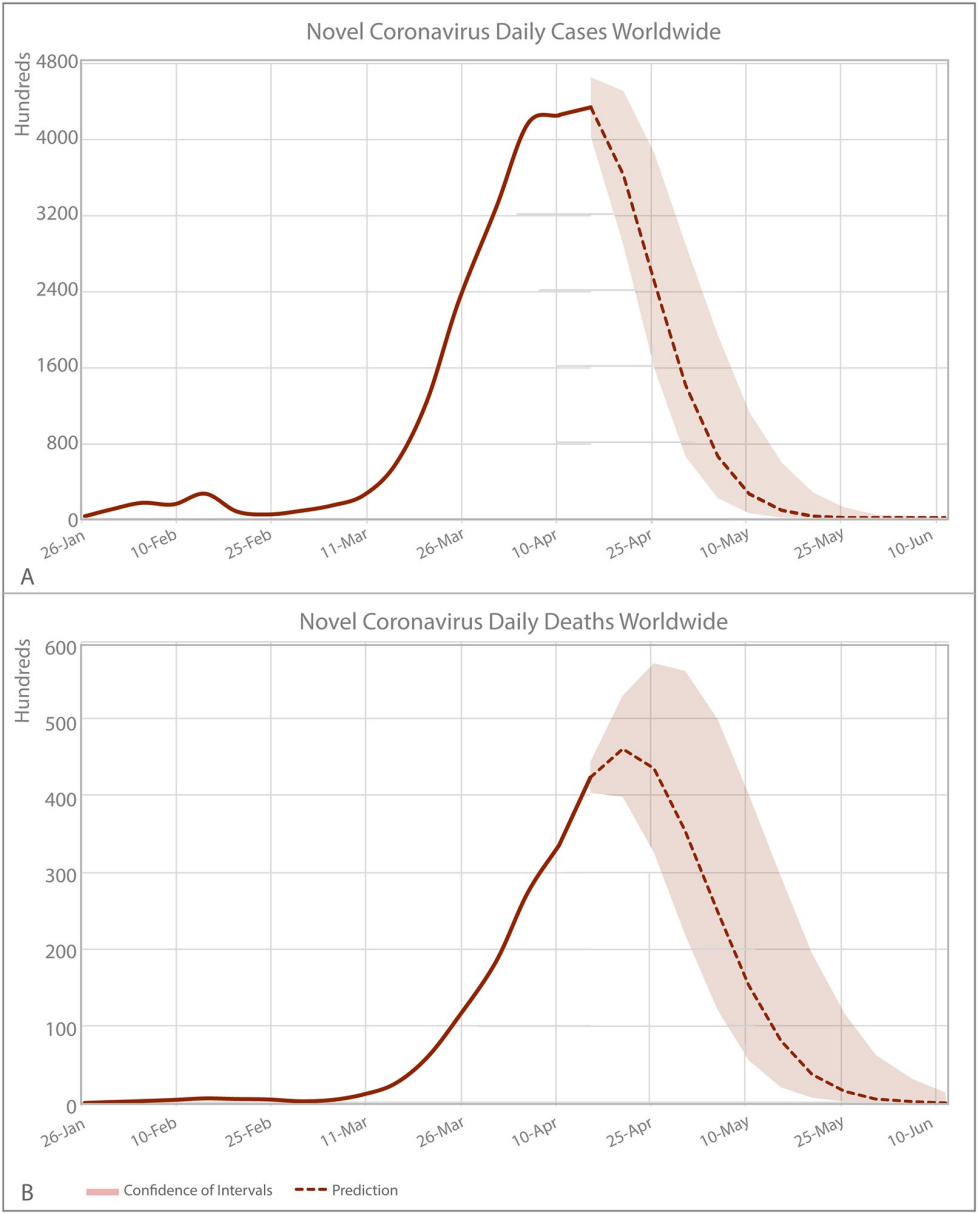
Worldwide COVID-19 new cases and deaths incidence predicted by Farr’s law. (A) New cases and (B) deaths incidence (and 95% CI). The calculations are based on worldwide data until April 10, 2020.

Worldwide we predict 1 284 553·6 (CI 95%, 935 337·5–1 988 290·9) of new cases (43·1% of the total cases) and 221 329·3 (CI 95%, 155 105·3–371 461·1) new deaths (68·1% of the total deaths) during the period after April 10 to June 10. The peak of new cases would reach around April 11 to 15th with approximately 432 4843·7 new cases (CI 95%, 400 294·6–464 672·7) and the peak of mortality around April 16 to 20th with approximately 46 051·7 deaths during this period (CI 95%, 39 846·2–52 870·2). Following a bell-shape curve, regardless neither of the new cases and new deaths reach zero until June 10, the lowest number of new cases would be around 1·2 (CI 95%, 0–321·3) new cases and 38 (CI 95%, 0·9–1 378·8) new deaths during the lowest peak on June 6th -10th. (Fig 3, S2 and S3 Tables).

**Fig 4:**
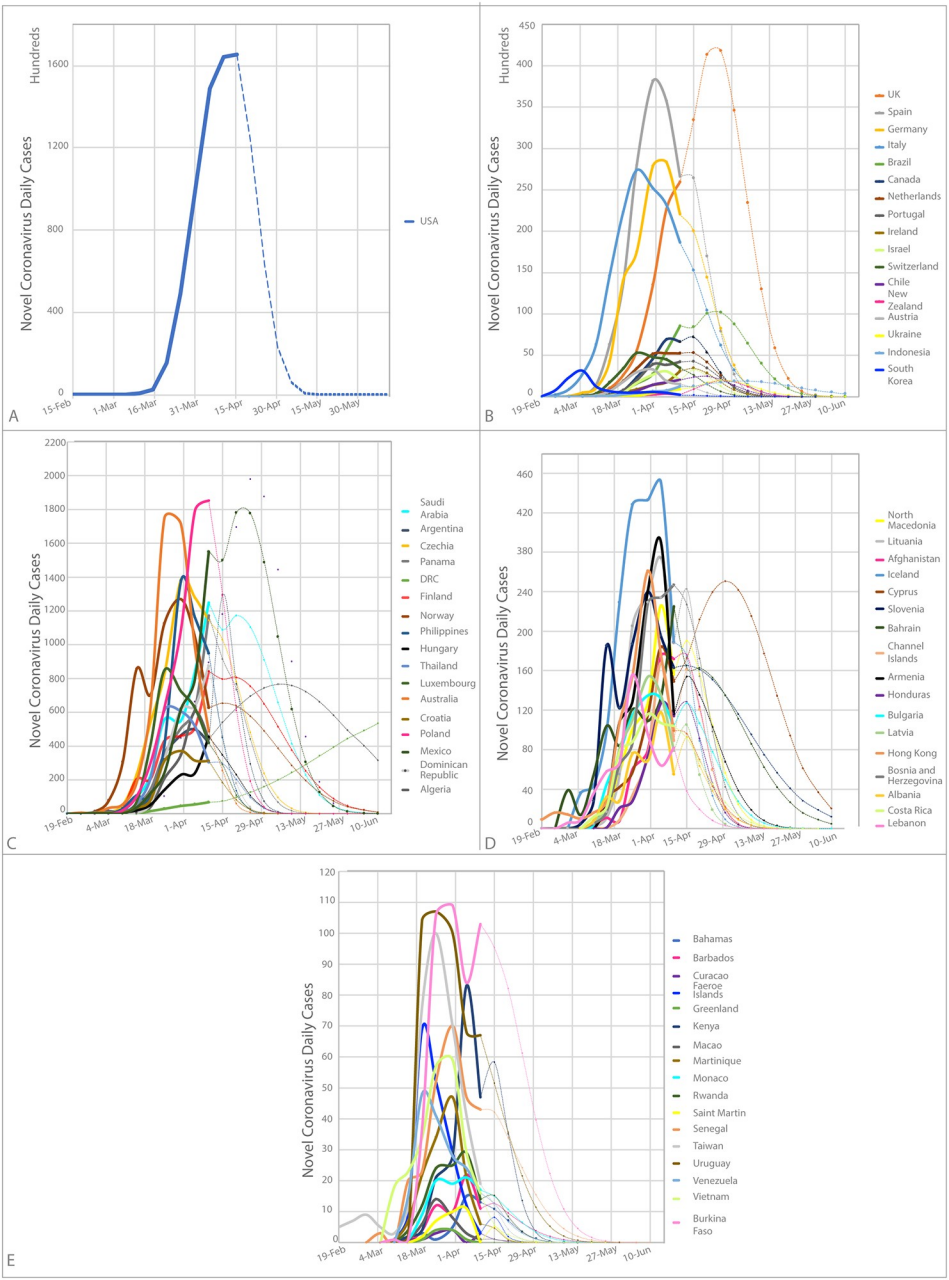
Prediction of new cases per country predicted by Farr’s law. A) USA prediction—the country with a higher incidence in the world. B) Prediction for quartile 1. C) Prediction for quartile 2. D) Prediction for quartile 3. E) Prediction for quartile 4. The quartile division is based on the number of new cases. Only 70 countries are included in this prediction analyses, due to the lack of data and failure to meet the assumptions criteria. The calculations are based on worldwide data up to April 10, 2020.

**Fig 5:**
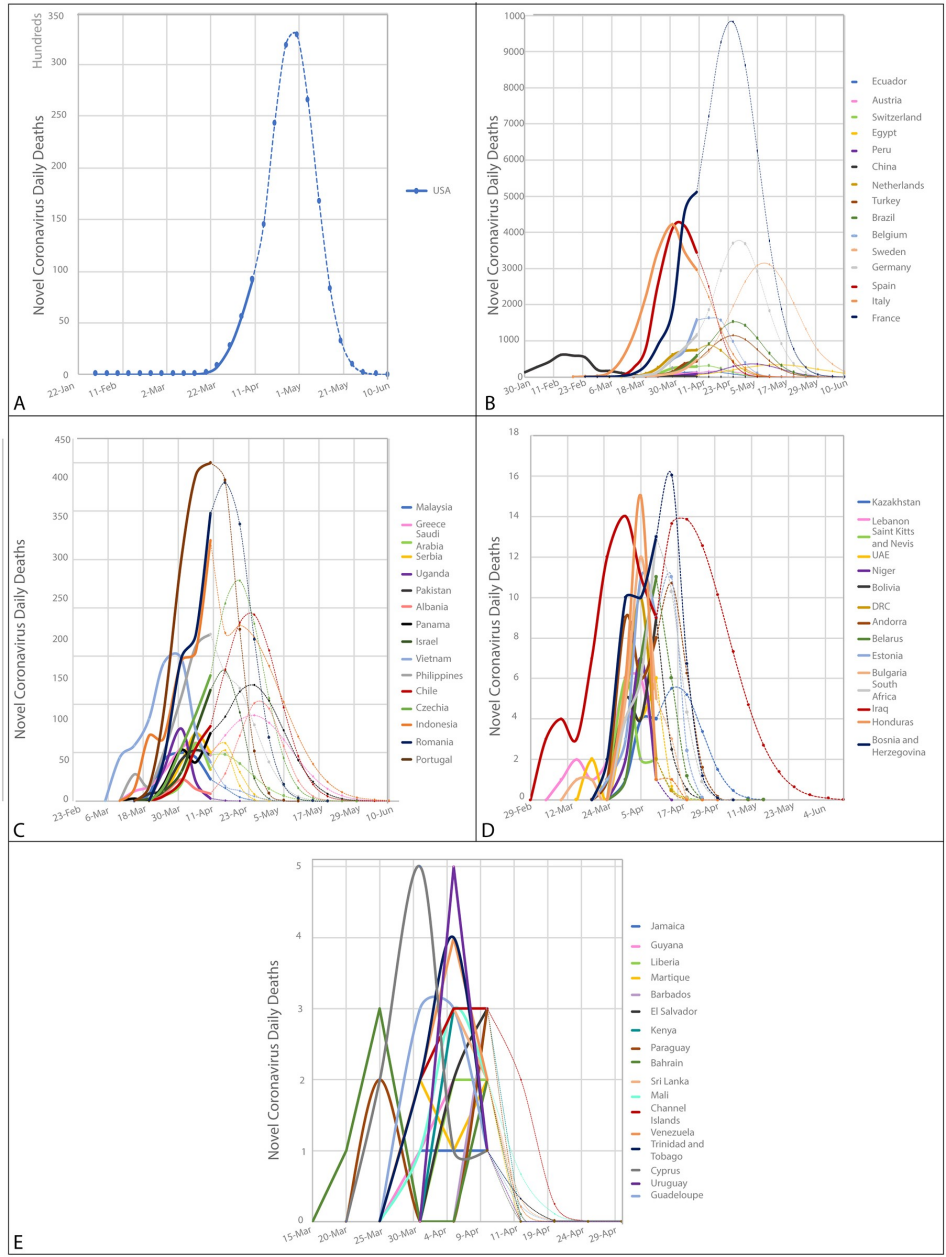
Prediction of new deaths per country predicted by Farr’s law. A) USA prediction—the country with a higher incidence in the world. B) Prediction for quartile 1. C) Prediction for quartile 2. D) Prediction for quartile 3. E) Prediction for quartile 4. The quartile division are based on the number of deaths. Only 68 countries are included in this prediction analyses, due to the lack of data and failure to meet the assumptions criteria. The calculations are based on worldwide data up to April 10, 2020.

Regarding the prediction of individual countries, we divided them into quartiles based on the number of daily cases and deaths trends (Figs 4 and 5). The highest quartile of new cases has a range of 1 863 to 165 364 daily cases and included 18 countries (56% from Europe, 22% from America, 17% from Asia and 6% from Oceania). The highest quartile for mortality includes values from 141 to 3 2867 daily deaths with 16 countries (10 [62%] from Europe, 4[25%] from America, 1[6%] from Asia, and 1[6%] from Africa). Regarding new cases, from 69 countries, 56 will reach zero, and 13 will continue beyond June 10, 2020 (see S2 Table). For new deaths, from 64 countries, 58 will reach zero deaths and 6 will still be continued beyond June 10, 2020 (in S3 Table). The countries with higher predicted cases are the USA, UK, and Spain, and higher predicted mortality are the USA, France, and Sweden (Figs 4 and 5 and S2 and S3 Tables).

#### Comparison with updated data

We found in our post-hoc comparison with updated data (June 10, 2020) that from the countries we predicted a higher number of cases and deaths worldwide (using data till April 10, 2020), 70% and 100% are actually among the first 20 countries with more cases and deaths, respectively, by June 2020. Our model predicted high dynamics in US, UK, Brazil, Italy, Spain, France (Table 1), and this was confirmed with current data. Additionally, we found 55 (26.2%) countries reported strict restriction strategies as part of political actions against the pandemic, the most common were stay-home policies, gathering restriction, and travel restrictions (S4 Table). However, there is important missing information for several countries in the available dataset (IHME).

Regarding the worldwide curve for new cases and deaths of Covid-19 in June 2020 (S1 Fig) showed a pseudo-normal distribution (negative kurtosis), with a steep slope by the second semester of March with a plateau by the beginning of April which last one month. By the beginning of May the curve started to rise again but gradually. Similarly, the curve of death had a steep slope during the last 10 days, from March reaching a peak by mid-April, and then a gradual descent until the end of May, where it reached a plateau.

By June 10, 36 countries (64.6%) of the 56 countries we predicted to be around no new cases before June 10 are decreasing or around zero new daily cases (Table 1). Moreover, as we predicted countries as New Zealand, Greenland, Macao, Saint Martin, and Faeroe Islands report no new cases for more than ten days up to June 10.

Concerning the 20 countries with high R1 for new Covid-19 cases (Table 2), we found that five countries (25%) were still having an increment on their curve with one of them (Uzbekistan) increasing a second wave. Two countries (10%) were in a plateau; for seven (35%), the descend on the curve was still not clear, while three (15%) has a clear descend and other three (15%) has not reported new cases for more than ten days. Among the three countries with the highest R1, Congo and Gabon are still reporting cases with a heterogeneous pattern, which hinders the determination of a clear dynamic on the curve. On the other hand, Angola reported its peak on June 10 and still in an accelerated phase of the pandemic (Table 2).

In the case of the countries with high R2 for cases, three countries (15%) had an increasing curve, one (5%) was in a plateau, three (15%) had a not clear descend, seven (35%) were descending and six (30%) had not reported cases for more than ten days. Among the countries with the highest R2, Angola and Bangladesh showed a clear increment on its curve, reporting more new cases on June 10 (Table 2).

## Discussion

Farr’s law is a simple arithmetical model that provides useful and important insights on epidemic dynamics. The findings from our modeling suggest that most of the countries over the world (76·43%) are in the early stage of the epidemic curve (phase A and B of our theoretical framework). The countries with higher epidemic dynamics (acceleration of cases and death numbers) are in Africa (around 40%) and had middle-income economies. Based on our model, the pandemic curve will reduce significantly until June 10, 2020, for both new cases and deaths, in the overall worldwide model and for 56 countries (in S2 and S3 Tables). The countries with higher predicted cases (adjusted for population) are USA, UK, and Spain, and higher predicted mortality are USA, France and Sweden; however, 60% of the countries could not enter to the predictive modeling due to lack of data or instability of R_2_ estimate (higher than 1).

The percentage of countries on phases A and B and with higher dynamics from low and middle-income sectors are higher (mainly from Africa and Latin America). This is a potential risk due to the limited health resources in those countries that could lead to a high rate of mortality and burden for the health system but also could generate a devastating socioeconomic, political, and inequality impact [19, 20]. Recent studies are reporting the lack of preparedness and high vulnerability of African countries against an eventual increase of COVID-19 cases [5]. Also, Moore et al. reported a ranking of countries based on the infectious disease vulnerability index [21], which considered a number of socioeconomic and health factors, several countries from the top of their list, such as Angola, Niger, Guinea, Congo, Togo, and Ivory Coast are in our ranking using the Farr’s ratios, indicating a higher epidemic dynamics in those countries, yet with a small number of cases, currently. Thus, this is a call to prioritize actions in those countries to intensify surveillance, to re-allocate resources, and to build healthcare capacities based on multi-nation collaboration [22] to limit onward transmission and to reduce the future impact on these regions.

Based on our prediction, the worldwide trend will reach values near to zero at the beginning in June, and approximately 56 countries (S2 Table) will reach values near zero before June 10, 2020. Compared with the current trend at June 10, 2020, we can see a pseudo-normal distribution with low kurtosis (more pronounced in the curve of new cases–S1 Fig). This could be explained by the heterogeneity of the clusters (countries) included in the model, with different pandemic start date, different socioeconomic characteristics, and public health and political actions against the pandemic; therefore, this produces potentially an overlap of multiple normal distributions curves. This also could be true for large countries with independent states such as USA (implementing multiple political actions and public health strategies at different moments) [23]. However, for more homogenous clusters (such as New Zealand, Australia, and some Asian countries), the predicted curves were accurate. Similarly, the prediction estimates were also accurate—most of the countries (70%) that we predicted a higher number of cases and deaths (till June 10) were confirmed in our post-hoc extraction, as well for the predicted countries with high dynamics (higher predicted R1 and R2). Thus, we should consider that our estimates using the Farr’s law depend on the precision of the reported data, the cluster heterogeneity, and the current acceleration (R_2_) of the epidemic dynamics (i.e. higher values of R_2_ produces an exponential function of the fitted values). Similar behavior was reported in previous studies [11, 12]. Although, Santillana et al. suggested a potential use of these higher R_2_ ratios, not to use them as predictive measures but rather as sentinel index for the change in epidemic dynamic, which could indicate the start of a new wave of cases [11].

Previous studies have reported behavior predictions for the COVID-19 pandemic; most of them focus on specific countries, such as China [24], Chile [25], Italy [24], France [24], and USA [23, 26]. The estimation of end date varies from May 12 (for Chile) to June 15 (for Italy), these dates from more complex models (most of them from a SIR model) are consistent with our predictions for those countries (Table 1), suggestion an acceptable accuracy to describe the epidemic dynamics with a simpler model. None of the previous studies used the Farr’s law to model the current pandemic behavior, and the available models reported prediction for high-income countries with better health system infrastructure and data registration; however, we could not identify published models from low- and middle-income countries, especially from Africa, those who are paradoxically more at risk due to high pandemic dynamic. Thus, Farr’s law approximation will be a valid option for scenarios with low resources and to identify countries at risk.

Moreover, it has been reported Farr’s law is an adequate model to assess the behavior of epidemics more than predict the exact number of cases accurately [2]. However, under certain assumptions (in epidemic phases with relative deceleration), its estimates are near to the SIR and IDEA models [11]. Besides, it seems to apply across different outbreaks types—because it relies on the intrinsic natural history of epidemics—and allow as to model fast with simple assumptions and limited data. The R_1_ and R_2_ ratios are variable across countries and epidemic phases, allowing us to classify the epidemic behavior over the world. Besides, it does seem that a higher R_1_ for mortality is associated with a high urban index and a higher number of deaths per 100,000 inhabitants, which is along with the literature on the impact of urbanization on the transmission of respiratory infection diseases [11]. Therefore, countries could also use R_1_ and R_2_ ratios to monitor the first deceleration phase (Phase B). Interestingly, the median R_2_ ratio is similar to the past AIDS epidemic reports [9], thus reflecting perhaps the behavior of an outbreak without immune protection.

The sociodemographic characteristics and the political actions against the pandemic are important factors within countries to describe pandemic behavior. From our model, we predicted 43 countries (Table 1) would reach near to zero in June. Most of them are middle to high-income countries, implement early and strict restriction policies; it seems no particular sociodemographic characteristics (population size, density, or proportion of older people) are predominant in these groups. These findings are aligned with previous studies showing the positive effects of strict restriction policies [27]. From our model and post-doc extraction, countries as New Zealand reached no new cases around May, while Australia has less than 20 new daily cases by June 10. The normality of these curves might be related to different explanations. First of all, both countries are high-income countries with a high Human Capital Index (0.8 and 0.7 respectively) that have invested in health since 1990 [28]. Germany, with 21.5% of the population more than 65 years old, has a smaller number of deaths per million people than other countries like US, Italy, Spain. All these countries established political and health social regulations during the pandemic and widespread testing even before reaching the peak. However, the key factors to successfully implement restrictive measurements could be adequate health literacy and socioeconomic equity [29]. For example, in our model, we found a high dynamic in Peru; it was one of the first countries in America to close its borders, established a strict lock-down with a curfew, and even provided financial aid for vulnerable people during the lock-down [30]. However, high socioeconomic disparities, great urban low-income conglomerations with limited health services accessibility, high levels of business informality [31], and even corruption have played an important role in jeopardizing the fight against the Covid-19 outbreak. Despite that, the Peruvian government’s quarantine policies might have prevented a major sanitary catastrophe considering the fragile and fragmented Peruvian public health system [32]. Indeed, our results suggested that restrictive measurements (social-distancing, restricting gatherings and non-essential travels) and widespread testing are critical to ending the ongoing COVID-19 pandemic. However, it is necessary to consider the sociodemographics and health literacy characteristics of the population to implement these measures in the mid- to long-term successfully.

One limitation in our modeling is the probable high rate of underreported cases all over the world, especially in low and middle economies either because of population size, weak health systems, geographical issues, inequity or lack of health access [33]. Additionally, the migration of people between countries is an important covariate for epidemic modeling [34] we did not include in our model due to data availability. In fact, this variable could contribute to underestimating the real dynamic; however, the utility for public health decision still valid as a similar limitation was founded in the previous influenza H1N1 pandemic [35]. Finally, another limitation is the potential heterogeneity on the criteria to identify cases in the Worldometer dataset, since they used a confirmation status based on public health policies and available test in each country. Therefore, there is a possibility of underreporting and false negatives cases [36], especially in low- and middle-income countries.

Finally, even though our model predicts a significant reduction of cases and deaths worldwide, a second wave of cases is possible. Currently, there are examples of countries with these patterns, such as South Korea and China, countries where the restriction strategies and political actions were applied early and rigorously. This highlights the importance of other factors such as viral reintroduction, particularly international importation from countries with higher epidemic dynamics, as well as a rebound of viral transmissibility due to the gradual resumption of economic activities and normal levels of social interaction [37]. In this scenario, our modeling approach could be a potential tool to assess the pandemic dynamics in a simple manner, especially in regions with already compromised health and socioeconomic systems.

In conclusion, to develop a global health perspective on a pandemic, the first step could be to use of simple modeling techniques to depict a broad global picture of the disease’s dynamics, that allow us to properly identify areas with high-risk due to high dynamic of the disease. Farr’s law seems to be a useful model to give an overview of COVID-19 pandemic dynamics. The regions with high dynamics are countries from Africa and Latin America. Thus, this is a call to urgently prioritize actions in those countries to intensify surveillance and re-allocate resources based on multi-nation collaboration to limit onward transmission and to reduce the future impact on these regions. Close monitoring of epidemic dynamics is needed to ensure correct worldwide policy interventions and to be prepared for an eventual COVID-19 second wave.

## Supporting information

S1 Table GATHER checklist. (DOC)

S2 Table Cases predicted. (XLSX)

S3 Table Deaths predicted. (XLSX)

S4 Table Restriction policies. (DOCX)

S1 Fig Worldwide COVID-19 new cases and deaths until June 10. (A) New daily cases and (B) new daily deaths incidence.
(TIF)

## References

[1] Organization WH. Coronavirus disease 2019 (COVID-19) Situation Report—83. World Health Organization, 2020 April 12, 2020. Report No.: Contract No.: 83.

[2] Wynants L, VC B Bonten MMJ, Collins GS, Debray TPA, De Vos M, Haller MC, et al Prediction models for diagnosis and prognosis of covid-19 infection: systematic review and critical appraisal. BMJ. 2020;369:M1328 10.1136/bmj.m1328. 32265220 PMC7222643

[3] Anastassopoulou C RL, Tsakris A, Siettos C. Data-based analysis, modelling and forecasting of the COVID-19 outbreak. PLoS ONE. 2020;15(3):e0230405 10.1371/journal.pone.0230405 32231374 PMC7108749

[4] Kucharski AJ, R T Diamond C, Liu Y, Edmunds J, Funk S, Eggo RM, Centre for Mathematical Modelling of Infectious Diseases COVID-19 working group. Early dynamics of transmission and control of COVID-19: a mathematical modelling study. Lancet Infect Dis. 2020 10.1016/S1473-3099(20)30144-4 32171059 PMC7158569

[5] Gilbert M, P G Pinotti F, Valdano E, Poletto C, Boëlle PY, D’Ortenzio E, et al Preparedness and vulnerability of African countries against importations of COVID-19: a modelling study. Lancet. 2020;396(10227):871–7. 10.1016/S0140-6736(20)30411-6 32087820 PMC7159277

[6] Hellewel J, A S, Gimma A, Bosse NI, Jarvis C, Russell TW, Munday JD, et al Feasibility of controlling COVID-19 outbreaks by isolation of cases and contacts. Lancet Glob Health. 2020;8(4):e488–e9. 10.1016/S2214-109X(20)30074-7 32119825 PMC7097845

[7] Jia Z L Z. Modelling COVID-19 transmission: from data to intervention. Lancet Infect Dis. 2020 10.1016/S1473-3099(20)30258-9 32246906 PMC7195346

[8] J B. Historical Note on Farr’s Theroy of The Epidemic. Br Med J. 1915;2(2850):250–2. 10.1136/bmj.2.2850.250 20767766 PMC2302838

[9] Bregnman DJ L A. Farr’s law applied to AIDS projections. JAMA. 1990;263(11):1522–5. 2308183

[10] Nsoesie EO, B J, Ramakrishnan N, Marathe MV. A systematic review of studies on forecasting the dynamics of influenza outbreaks. Influenza Other Respir Viruses. 2013;8(3):309–16. 10.1111/irv.12226 24373466 PMC4181479

[11] Santillana M, T A, Nasserie T, Fine P, Champredon D, Chindelevitch L, Dushoff J, et al Relatedness of the incidence decay with exponential adjustment (IDEA) model, "Farr’s law" and SIR compartmental difference equation models. Infect Dis Model. 2018;3:1–12. 10.1016/j.idm.2018.03.001 30839910 PMC6326218

[12] Darakjy S, B J, DiMaggio CJ, Li Guohua. Applying Farr’s Law to project the drug overdose mortality epidemic in the United States. Inj Epidemiol. 2014;1(31). 10.1186/s40621-014-0031-2 27747664 PMC5005643

[13] Worldometer. COVID-19 CORONAVIRUS PANDEMIC 2020 [cited 2020]. https://www.worldometers.info/coronavirus/.

[14] Otto-Zimmermann K. From Rio to Rio+ 20: the changing role of local governments in the context of current global governance. Local Environment. 2012;17(5):511–6.

[15] Covid C. Dashboard by the Center for Systems Science and Engineering (CSSE) at Johns Hopkins University (JHU). 2020.

[16] Bank TW. 2019 [cited 2020]. https://data.worldbank.org/.

[17] Institute for Health Metrics and Evaluation. COVID-19 projections. Seattle, WA: IHME—University of Washington; 2020 [cited 2020 June 06]. https://covid19.healthdata.org/projections.

[18] Stevens GA A L, Black RE, Boerman JT, Collins GS, Ezzati M, Grove JT, et al The GATHER Working Group. Guidelines for Accurate and Transparent Health Estimates Reporting: the GATHER statement. Lancet. 2016;388(10062):PE19–E23. 10.1016/S0140-6736(16)30388-9 27371184

[19] Quinn SC K S. Health Inequalities and Infectious Disease Epidemics: A Challenge for Global Health Security. Biosecur Bioterr. 2014;12(5):263–73. 10.1089/bsp.2014.0032 25254915 PMC4170985

[20] Hopman J A B, Mehtar S. Managing COVID-19 in Low- and Middle-Income Countries. JAMA. 2020 Epub ahead of print. 10.1001/jama.2020.4169 32176764

[21] Moore M GB, Okunogbe A, Paul C. Identifying Future Disease Hot Spots. California: RAND Corporation; 2016. 10.7249/rhq-06-03-05 PMC5568150 28845357

[22] Nkengasong JN M W. Looming threat of COVID-19 infection in Africa: act collectively, and fast. Lancet. 2020;395(10227):P841–2. 10.1016/S0140-6736(20)30464-5 32113508 PMC7124371

[23] Velásquez RMA, Lara JVM. Forecast and evaluation of COVID-19 spreading in USA with Reduced-space Gaussian process regression. Chaos, Solitons & Fractals. 2020:109924 10.1016/j.chaos.2020.109924 32501372 PMC7242925

[24] Fanelli D, Piazza F. Analysis and forecast of COVID-19 spreading in China, Italy and France. Chaos, Solitons & Fractals. 2020;134:109761 10.1016/j.chaos.2020.109761 32308258 PMC7156225

[25] Guerrero-Nancuante C, Manríquez R. Proyección epidemiológica de COVID-19 en Chile basado en el modelo SEIR generalizado y el concepto de recuperado. Medwave. 2020;20(04). 10.5867/medwave.2020.04.7898 32469853

[26] Chen D-G, Chen X, Chen JK. Reconstructing and forecasting the COVID-19 epidemic in the United States using a 5-parameter logistic growth model. Global Health Research and Policy. 2020;5:1–7. 10.1186/s41256-019-0129-8 32435695 PMC7225094

[27] Giordano G, Blanchini F, Bruno R, Colaneri P, Di Filippo A, Di Matteo A, et al Modelling the COVID-19 epidemic and implementation of population-wide interventions in Italy. Nature Medicine. 2020:1–6. 10.1038/s41591-019-0740-8 32322102 PMC7175834

[28] Fullman N, Yearwood J, Abay SM, Abbafati C, Abd-Allah F, Abdela J, et al Measuring performance on the Healthcare Access and Quality Index for 195 countries and territories and selected subnational locations: a systematic analysis from the Global Burden of Disease Study 2016. The Lancet. 2018;391(10136):2236–71. 10.1016/S0140-6736(18)30994-2 29893224 PMC5986687

[29] Paakkari L, Okan O. COVID-19: health literacy is an underestimated problem. The Lancet Public Health. 2020;5(5):e249–e50. 10.1016/S2468-2667(20)30086-4 32302535 PMC7156243

[30] Maxmen A. How poorer countries are scrambling to prevent a coronavirus disaster. Nature. 2020;580(7802):173 10.1038/d41586-020-00983-9 32242110

[31] Romero TH, Reys A. 243. Empobrecimiento de los hogares y cambios en el abastecimiento de alimentos por la COVID-19 en Lima, Perú. Ar@ cne. 2020;24.

[32] Sanchez-Moreno F. The national health system in Peru. Revista peruana de medicina experimental y salud publica. 2014;31(4):747–53. 25597729

[33] Tuite AR, N V, Rees E, Fisman D. Estimation of COVID-19 outbreak size in Italy. Lancet Infect Dis. 2020 Epub Ahead of print. 10.1016/S1473-3099(20)30227-9 32199494 PMC7271205

[34] Greenaway C, Gushulak BD. Pandemics, migration and global health security Handbook on migration and security: Edward Elgar Publishing; 2017.

[35] Reed C, A F, Swerdlow DL, Lipsitch M, Meltzer MI, Jernigan D, Finelli L. Estimates of the Prevalence of Pandemic (H1N1) 2009, United States, April–July 2009. Emerg Infect Dis. 2009;15(12):2004–7. 10.3201/eid1512.091413 19961687 PMC3375879

[36] Tang Y-W, Schmitz JE, Persing DH, Stratton CW. Laboratory diagnosis of COVID-19: current issues and challenges. Journal of clinical microbiology. 2020;58(6). 10.1128/JCM.00512-20 32245835 PMC7269383

[37] Kissler SM, T C, Goldstein E, Grad YH, Lipsitch. Projecting the transmission dynamics of SARS-CoV-2 through the postpandemic period. Science. 2020 10.1126/science.abb5793 32291278 PMC7164482

