# Supplementary material for: COVID-19 pandemic and Farr’s law: A global comparison and prediction of outbreak acceleration and deceleration rates
Source: PLoS One. 2020 Sep 17;15(9):e0239175. doi: 10.1371/journal.pone.0239175 (PMC7498003; doi:10.1371/journal.pone.0239175)
Supplement: S4 Table — (DOCX) [file pone.0239175.s004.docx]

| **Table S4 - Restriction policies** | | | | | | | | | | | |
| --- | --- | --- | --- | --- | --- | --- | --- | --- | --- | --- | --- |
| Country | Subregion | Travel Restriction Start Date | Travel Restriction End Date | Stay Home Start Day | Stay at Home End Day | Educational Facts Start Day | Educational Facts End Day | Any Gathering Restriction Start Day | Any Gathering Restriction End Day | All Non-Essential Bussiness Restriction Start Day | All Non-Essential Bussiness Restriction End Day |
| **Argentina** |  | 3/20/20 | 4/27/20 | 3/20/20 |  | 3/16/20 |  | 3/15/20 |  | 3/20/20 |  |
| **Austria** |  |  |  | 3/16/20 | 5/01/20 | 3/16/20 |  | 3/10/20 |  | 3/16/20 | 4/13/20 |
| **Belgium** |  |  |  | 3/18/20 |  | 3/14/20 |  | 3/13/20 |  | 3/18/20 | 5/11/20 |
| **Bolivia** |  | 3/27/20 |  | 3/26/20 |  | 3/12/20 |  | 3/12/20 |  | 3/20/20 |  |
| **Brazil** |  |  |  |  |  | 3/24/20 |  |  |  |  |  |
|  | Acre |  |  |  |  | 3/16/20 |  | 3/20/20 |  | 3/17/20 |  |
|  | Alagoas |  |  |  |  | 3/17/20 |  | 3/16/20 |  | 4/07/20 | 5/06/20 |
|  | Amazonas | 4/06/20 |  |  |  | 3/16/20 |  | 3/31/20 |  | 3/24/20 |  |
|  | Bahia |  |  |  |  | 3/17/20 |  | 3/27/20 |  |  |  |
|  | Espírito Santo |  |  |  |  | 3/16/20 |  | 3/17/20 |  | 3/20/20 |  |
|  | Mato Grosso |  |  | 3/31/20 |  | 3/23/20 |  | 3/16/20 |  | 3/26/20 | 4/22/20 |
|  | Mato Grosso do Sul |  |  |  |  | 3/23/20 |  | 3/16/20 |  |  |  |
|  | Minas Gerais |  |  |  |  | 3/20/20 |  | 3/20/20 |  |  |  |
|  | Paraiba |  |  |  |  | 3/19/20 |  | 3/17/20 |  |  |  |
|  | Parana |  |  |  |  | 3/20/20 |  | 3/16/20 |  | 3/21/20 |  |
|  | Pernambuco |  |  |  |  | 3/18/20 |  |  |  | 3/20/20 |  |
|  | Piaui |  |  |  |  | 3/16/20 |  | 3/16/20 |  | 3/23/20 |  |
|  | Rio de Janeiro |  |  | 3/30/20 |  | 3/13/20 |  | 3/13/20 |  |  |  |
|  | Rio Grande do Norte |  |  |  |  | 3/24/20 |  | 4/01/20 |  | 4/01/20 |  |
|  | Rio Grande do Sul |  |  |  |  | 4/01/20 |  | 3/19/20 |  | 4/01/20 | 4/08/20 |
|  | Rodonia |  |  |  |  | 3/17/20 |  | 3/20/20 |  | 3/20/20 |  |
|  | Roraima |  |  |  |  | 3/17/20 |  | 3/16/20 |  | 3/27/20 |  |
|  | Santa Catarina |  |  |  |  | 3/19/20 |  | 3/17/20 |  | 3/17/20 | 4/13/20 |
|  | Sao Paulo |  |  |  |  | 3/23/20 |  | 3/14/20 |  | 3/24/20 |  |
|  | Sergipe |  |  |  |  | 3/16/20 |  | 3/16/20 |  | 3/24/20 | 4/16/20 |
|  | Tocantins |  |  |  |  | 3/18/20 |  | 3/21/20 |  |  |  |
| **Bulgaria** |  | 3/21/20 |  | 3/17/20 | 5/03/20 | 3/13/20 |  | 3/13/20 |  | 3/13/20 | 5/06/20 |
| **Canada** |  |  |  |  |  |  |  |  |  |  |  |
|  | Alberta |  |  |  |  | 3/16/20 |  | 3/12/20 |  |  |  |
|  | British Columbia |  |  |  |  | 3/17/20 |  | 3/16/20 |  |  |  |
|  | Canada |  |  |  |  | 3/23/20 |  | 3/23/20 |  |  |  |
|  | Ontario |  |  |  |  | 3/16/20 |  | 3/16/20 |  | 4/05/20 | 5/04/20 |
|  | Quebec |  |  | 3/21/20 |  | 3/16/20 |  | 3/12/20 |  | 3/23/20 | 5/11/20 |
| **Chile** |  |  |  |  |  | 3/16/20 |  | 3/16/20 |  |  |  |
| **Colombia** |  | 3/25/20 |  | 3/25/20 |  | 3/16/20 |  | 3/16/20 |  | 3/25/20 | 4/27/20 |
| **Croatia** |  | 3/23/20 |  | 3/17/20 | 5/11/20 | 3/16/20 |  | 3/09/20 |  | 3/19/20 | 4/27/20 |
| **Cuba** |  | 4/11/20 |  |  |  | 3/24/20 |  | 3/20/20 |  |  |  |
| **Cyprus** |  |  |  | 3/24/20 | 5/04/20 | 3/13/20 |  | 3/24/20 |  | 3/24/20 | 5/04/20 |
| **Czechia** |  |  |  | 3/16/20 | 4/20/20 | 3/10/20 |  | 3/10/20 |  | 3/14/20 | 4/20/20 |
| **Denmark** |  |  |  |  |  | 3/16/20 |  | 3/18/20 |  |  |  |
| **Dominican Republic** |  | 3/19/20 | 4/13/20 |  |  | 3/19/20 |  | 3/25/20 |  | 3/19/20 | 5/20/20 |
| **Ecuador** |  | 3/18/20 | 6/01/20 | 3/17/20 |  | 3/13/20 |  | 3/15/20 |  | 3/17/20 |  |
| **Egypt** |  |  |  |  |  | 3/15/20 |  | 3/09/20 |  |  |  |
| **Estonia** |  |  |  |  |  | 3/16/20 |  | 3/13/20 |  |  |  |
| **Finland** |  | 3/25/20 |  |  |  | 3/18/20 |  | 3/12/20 |  | 4/04/20 | 6/01/20 |
| **France** |  | 3/17/20 | 5/11/20 | 3/18/20 | 5/11/20 | 3/13/20 | 5/25/20 | 3/04/20 |  | 3/15/20 | 5/11/20 |
| **Germany** |  |  |  |  |  |  |  |  |  |  |  |
|  | Baden-W√ºrttemberg |  |  | 3/21/20 | 5/11/20 | 3/17/20 |  | 3/21/20 |  | 3/21/20 | 4/20/20 |
|  | Bavaria |  |  | 3/21/20 | 5/06/20 | 3/16/20 |  | 3/21/20 |  | 3/21/20 | 4/27/20 |
|  | Berlin |  |  | 3/23/20 | 5/09/20 | 3/23/20 |  | 3/23/20 |  | 3/23/20 | 4/22/20 |
|  | Brandenburg |  |  | 3/17/20 | 5/09/20 | 3/18/20 |  | 3/17/20 |  | 3/17/20 | 4/22/20 |
|  | Bremen |  |  | 3/22/20 | 5/14/20 | 3/16/20 |  | 3/22/20 |  | 3/20/20 | 4/20/20 |
|  | Hamburg |  |  | 3/22/20 | 5/04/20 | 3/16/20 |  | 3/22/20 |  |  |  |
|  | Hesse |  |  | 3/22/20 | 5/09/20 | 3/16/20 |  | 3/22/20 |  |  |  |
|  | Lower Saxony |  |  | 3/23/20 | 5/11/20 | 3/16/20 |  | 3/23/20 |  | 3/27/20 | 4/20/20 |
|  | Mecklenburg-Vorpommern |  |  | 3/23/20 | 5/07/20 | 3/16/20 |  | 3/23/20 |  | 3/18/20 | 4/20/20 |
|  | North Rhine-Westphalia |  |  | 3/23/20 | 5/11/20 | 3/16/20 |  | 3/23/20 |  | 3/23/20 | 4/20/20 |
|  | Rhineland-Palatinate |  |  | 3/22/20 | 5/13/20 | 3/16/20 |  | 3/22/20 |  | 3/23/20 | 4/20/20 |
|  | Saarland |  |  | 3/21/20 | 5/18/20 | 3/16/20 |  | 3/21/20 |  |  |  |
|  | Saxony |  |  | 3/23/20 | 4/20/20 | 3/23/20 |  | 3/23/20 |  | 3/23/20 | 4/20/20 |
|  | Saxony-Anhalt |  |  | 3/22/20 | 5/04/20 | 3/16/20 |  | 3/22/20 |  | 3/24/20 | 4/20/20 |
|  | Schleswig-Holstein |  |  | 3/24/20 | 5/09/20 | 3/16/20 |  | 3/24/20 |  | 3/24/20 | 4/20/20 |
|  | Thuringia |  |  | 3/22/20 | 5/13/20 | 3/17/20 |  | 3/22/20 |  |  |  |
| **Greece** |  | 3/23/20 |  | 3/23/20 | 5/04/20 | 3/11/20 |  | 3/08/20 |  | 3/22/20 | 5/04/20 |
| **Honduras** |  | 3/16/20 |  | 3/19/20 |  | 3/13/20 |  | 3/14/20 |  | 3/16/20 |  |
| **Hungary** |  |  |  | 3/28/20 | 5/18/20 | 3/16/20 |  | 3/12/20 |  | 3/16/20 | 5/18/20 |
| **Iceland** |  |  |  |  |  | 3/16/20 | 5/04/20 | 3/16/20 |  |  |  |
| **Ireland** |  |  |  | 3/27/20 | 5/18/20 | 3/12/20 |  | 3/12/20 |  | 3/24/20 | 5/18/20 |
| **Israel** |  |  |  | 3/25/20 | 4/19/20 | 3/12/20 |  | 3/04/20 |  | 3/25/20 | 5/03/20 |
| **Italy** |  |  |  |  |  |  |  |  |  |  |  |
|  | Abruzzo | 3/22/20 | 5/04/20 | 3/11/20 | 6/03/20 | 3/05/20 |  | 3/11/20 |  | 3/11/20 | 4/14/20 |
|  | Basilicata | 3/22/20 | 5/04/20 | 3/11/20 | 6/03/20 | 3/05/20 |  | 3/11/20 |  | 3/11/20 | 4/14/20 |
|  | Calabria | 3/22/20 | 5/04/20 | 3/11/20 | 6/03/20 | 3/05/20 |  | 3/11/20 |  | 3/11/20 | 4/14/20 |
|  | Campania | 3/22/20 | 5/04/20 | 3/11/20 | 6/03/20 | 3/05/20 |  | 3/11/20 |  | 3/11/20 | 4/14/20 |
|  | Emilia-Romagna | 3/22/20 | 5/04/20 | 3/11/20 | 6/03/20 | 3/01/20 |  | 3/07/20 |  | 3/11/20 | 4/14/20 |
|  | Friuli-Venezia Giulia | 3/22/20 | 5/04/20 | 3/11/20 | 6/03/20 | 3/05/20 |  | 3/11/20 |  | 3/11/20 | 4/14/20 |
|  | Lazio | 3/22/20 | 5/04/20 | 3/11/20 | 6/03/20 | 3/05/20 |  | 3/11/20 |  | 3/11/20 | 4/14/20 |
|  | Liguria | 3/22/20 | 5/04/20 | 3/11/20 | 6/03/20 | 3/05/20 |  | 3/11/20 |  | 3/11/20 | 4/14/20 |
|  | Lombardia | 3/08/20 | 5/04/20 | 3/08/20 | 6/03/20 | 3/01/20 |  | 2/22/20 |  | 3/08/20 | 4/14/20 |
|  | Marche | 3/22/20 | 5/04/20 | 3/11/20 | 6/03/20 | 3/05/20 |  | 3/07/20 |  | 3/11/20 | 4/14/20 |
|  | Molise | 3/22/20 | 5/04/20 | 3/11/20 | 6/03/20 | 3/05/20 |  | 3/11/20 |  | 3/11/20 | 4/14/20 |
|  | Piemonte | 3/22/20 | 5/04/20 | 3/11/20 | 6/03/20 | 3/05/20 |  | 3/07/20 |  | 3/11/20 | 4/14/20 |
|  | Provincia autonoma di Bolzano | 3/22/20 | 5/04/20 | 3/11/20 | 6/03/20 | 3/05/20 |  | 3/11/20 |  | 3/11/20 | 4/14/20 |
|  | Provincia autonoma di Trento | 3/22/20 | 5/04/20 | 3/11/20 | 6/03/20 | 3/05/20 |  | 3/11/20 |  | 3/11/20 | 4/14/20 |
|  | Puglia | 3/22/20 | 5/04/20 | 3/11/20 | 6/03/20 | 3/05/20 |  | 3/11/20 |  | 3/11/20 | 4/14/20 |
|  | Sardegna | 3/22/20 | 5/04/20 | 3/11/20 | 6/03/20 | 3/05/20 |  | 3/11/20 |  | 3/11/20 | 4/14/20 |
|  | Sicilia | 3/22/20 | 5/04/20 | 3/11/20 | 6/03/20 | 3/05/20 |  | 3/11/20 |  | 3/11/20 | 4/14/20 |
|  | Toscana | 3/22/20 | 5/04/20 | 3/11/20 | 6/03/20 | 3/05/20 |  | 3/11/20 |  | 3/11/20 | 4/14/20 |
|  | Umbria | 3/22/20 | 5/04/20 | 3/11/20 | 6/03/20 | 3/05/20 |  | 3/11/20 |  | 3/11/20 | 4/14/20 |
|  | Valle d'Aosta | 3/22/20 | 5/04/20 | 3/11/20 | 6/03/20 | 3/05/20 |  | 3/11/20 |  | 3/11/20 | 4/14/20 |
|  | Veneto | 3/22/20 | 5/04/20 | 3/11/20 | 6/03/20 | 3/01/20 |  | 2/22/20 |  | 3/11/20 | 4/14/20 |
| **Japan** |  |  |  |  |  | 3/02/20 |  |  |  |  |  |
| **Latvia** |  |  |  |  |  | 3/12/20 |  | 3/13/20 |  |  |  |
| **Lithuania** |  |  |  | 3/15/20 | 4/29/20 | 3/16/20 |  | 3/15/20 |  | 3/15/20 | 4/15/20 |
| **Luxembourg** |  |  |  |  |  | 3/16/20 |  | 3/13/20 |  | 3/18/20 | 4/20/20 |
| **Malaysia** |  | 3/18/20 |  | 3/18/20 |  | 3/18/20 |  | 3/13/20 |  | 3/18/20 | 5/04/20 |
| **Mexico** |  |  |  |  |  | 3/23/20 |  | 3/24/20 |  | 4/04/20 |  |
|  | Aguascalientes |  |  |  |  | 3/19/20 |  | 3/13/20 |  | 3/30/20 |  |
|  | Baja California |  |  |  |  | 3/19/20 |  | 3/18/20 |  | 3/30/20 |  |
|  | Baja California Sur |  |  |  |  | 3/19/20 |  | 3/16/20 |  | 3/30/20 |  |
|  | Campeche |  |  |  |  | 3/18/20 |  | 3/19/20 |  | 3/30/20 |  |
|  | Chiapas |  |  |  |  | 3/22/20 |  | 3/15/20 |  | 3/30/20 |  |
|  | Chihuahua |  |  |  |  | 3/23/20 |  | 3/25/20 |  | 3/24/20 |  |
|  | Coahuila |  |  |  |  | 3/19/20 |  | 3/18/20 |  | 3/30/20 |  |
|  | Colima |  |  |  |  | 3/16/20 |  | 3/16/20 |  | 3/18/20 |  |
|  | Distrito Federal |  |  |  |  | 3/12/20 |  | 3/12/20 |  | 3/19/20 |  |
|  | Durango |  |  | 4/19/20 |  | 3/22/20 |  | 4/02/20 |  | 3/30/20 |  |
|  | Guanajuato |  |  |  |  | 3/16/20 |  | 3/24/20 |  | 3/30/20 |  |
|  | Guerrero |  |  |  |  | 3/22/20 |  | 3/24/20 |  | 4/04/20 |  |
|  | Hidalgo |  |  |  |  | 3/22/20 |  | 3/24/20 |  | 3/30/20 |  |
|  | Jalisco |  |  | 4/19/20 |  | 3/16/20 |  | 3/24/20 |  | 3/30/20 |  |
|  | Mexico City |  |  |  |  | 3/19/20 |  | 3/16/20 |  | 4/01/20 |  |
|  | Michoacán de Ocampo |  |  |  |  | 3/23/20 |  | 3/15/20 |  | 3/30/20 |  |
|  | Morelos |  |  |  |  | 3/17/20 |  | 3/20/20 |  | 3/30/20 |  |
|  | Nayarit |  |  |  |  | 3/22/20 |  | 3/15/20 |  | 3/30/20 |  |
|  | Nuevo León |  |  |  |  | 3/22/20 |  | 3/23/20 |  | 3/30/20 |  |
|  | Oaxaca |  |  |  |  | 3/22/20 |  | 3/12/20 |  | 3/30/20 |  |
|  | Puebla |  |  |  |  | 3/22/20 |  | 3/23/20 |  | 3/30/20 |  |
|  | Querataro |  |  |  |  | 3/15/20 |  | 3/13/20 |  | 3/30/20 |  |
|  | Quintana Roo |  |  |  |  | 3/22/20 |  | 3/24/20 |  | 3/30/20 |  |
|  | San Luis Potos√≠ |  |  |  |  | 3/22/20 |  | 3/20/20 |  | 3/30/20 |  |
|  | Sinaloa |  |  |  |  | 3/19/20 |  | 3/20/20 |  | 3/30/20 |  |
|  | Sonora |  |  | 4/12/20 |  | 3/16/20 |  | 3/17/20 |  | 3/30/20 |  |
|  | Tabasco |  |  |  |  | 3/22/20 |  | 3/14/20 |  | 4/01/20 |  |
|  | Tamaulipas |  |  |  |  | 3/16/20 |  | 3/13/20 |  | 3/30/20 |  |
|  | Tlaxcala |  |  |  |  | 3/15/20 |  | 3/27/20 |  | 3/23/20 |  |
|  | Veracruz de Ignacio de la Llave |  |  |  |  | 3/16/20 |  | 3/16/20 |  | 3/30/20 |  |
|  | Yucatan |  |  |  |  | 3/16/20 |  | 3/26/20 |  | 3/30/20 |  |
|  | Zacatecas |  |  |  |  | 3/23/20 |  | 3/15/20 |  | 3/30/20 |  |
| **Netherlands** |  |  |  |  |  | 3/15/20 | 6/15/20 | 3/10/20 |  |  |  |
| **Norway** |  |  |  |  |  | 3/12/20 | 5/15/20 | 3/12/20 |  |  |  |
| **Panama** |  | 3/13/20 | 6/01/20 | 3/25/20 | 6/01/20 | 3/16/20 |  | 3/25/20 |  | 3/25/20 | 5/13/20 |
| **Peru** |  |  |  | 3/16/20 |  | 3/16/20 |  | 3/12/20 |  | 3/16/20 | 5/01/20 |
| **Phillipines** |  |  |  |  |  | 3/16/20 |  |  |  |  |  |
| **Poland** |  |  |  | 3/24/20 | 4/20/20 | 3/12/20 | 5/24/20 | 3/10/20 |  |  |  |
| **Portugal** |  | 4/09/20 |  | 3/19/20 | 5/04/20 | 3/16/20 |  | 3/19/20 |  | 3/19/20 | 5/04/20 |
| **Republic of Korea** |  |  |  |  |  | 3/02/20 | 6/08/20 |  |  |  |  |
| **Republic of Moldova** |  |  |  | 3/25/20 |  | 3/11/20 |  | 3/10/20 |  | 3/16/20 |  |
| **Romania** |  |  |  | 3/23/20 | 5/15/20 | 3/11/20 |  | 3/06/20 |  | 3/21/20 | 5/15/20 |
| **Russian Federation** |  |  |  |  |  | 3/23/20 |  |  |  | 3/28/20 | 5/12/20 |
| **Serbia** |  | 3/22/20 | 5/04/20 | 3/17/20 | 5/07/20 | 3/16/20 |  | 3/11/20 |  | 3/16/20 |  |
| **Slovakia** |  | 4/08/20 | 4/14/20 |  |  | 3/12/20 |  | 3/12/20 |  | 3/16/20 | 3/30/20 |
| **Slovenia** |  | 3/16/20 | 4/30/20 |  |  | 3/16/20 |  | 3/12/20 |  | 3/15/20 | 4/20/20 |
| **Spain** |  |  |  |  |  |  |  |  |  |  |  |
|  | Andalucia |  |  | 3/15/20 | 5/18/20 | 3/14/20 |  | 3/15/20 |  | 3/15/20 | 4/13/20 |
|  | Aragon |  |  | 3/15/20 | 5/11/20 | 3/14/20 |  | 3/15/20 |  | 3/15/20 | 4/13/20 |
|  | Asturias |  |  | 3/15/20 | 5/11/20 | 3/14/20 |  | 3/15/20 |  | 3/15/20 | 4/13/20 |
|  | Balearic Islands |  |  | 3/15/20 | 5/11/20 | 3/14/20 |  | 3/15/20 |  | 3/15/20 | 4/13/20 |
|  | Basque Country |  |  | 3/15/20 | 5/11/20 | 3/14/20 |  | 3/15/20 |  | 3/15/20 | 4/13/20 |
|  | Canary Islands |  |  | 3/15/20 | 5/11/20 | 3/14/20 |  | 3/15/20 |  | 3/15/20 | 4/13/20 |
|  | Cantabria |  |  | 3/15/20 | 5/11/20 | 3/14/20 |  | 3/15/20 |  | 3/15/20 | 4/13/20 |
|  | Castile and Leon |  |  | 3/15/20 | 5/25/20 | 3/14/20 |  | 3/15/20 |  | 3/15/20 | 4/13/20 |
|  | Castilla-La Mancha |  |  | 3/15/20 | 5/18/20 | 3/14/20 |  | 3/15/20 |  | 3/15/20 | 4/13/20 |
|  | Catalonia |  |  | 3/15/20 | 5/25/20 | 3/14/20 |  | 3/11/20 |  | 3/15/20 | 5/25/20 |
|  | Ceuta |  |  | 3/15/20 | 5/11/20 | 3/14/20 |  | 3/15/20 |  | 3/15/20 | 4/13/20 |
|  | Community of Madrid |  |  | 3/15/20 | 5/25/20 | 3/11/20 |  | 3/15/20 |  | 3/13/20 | 4/13/20 |
|  | Extremadura |  |  | 3/15/20 | 5/11/20 | 3/14/20 |  | 3/15/20 |  | 3/15/20 | 4/13/20 |
|  | Galicia |  |  | 3/15/20 | 5/11/20 | 3/14/20 |  | 3/15/20 |  | 3/15/20 | 4/13/20 |
|  | La Rioja |  |  | 3/15/20 | 5/11/20 | 3/14/20 |  | 3/15/20 |  | 3/15/20 | 4/13/20 |
|  | Murcia |  |  | 3/15/20 | 5/11/20 | 3/14/20 |  | 3/15/20 |  | 3/15/20 | 4/13/20 |
|  | Valencian Community |  |  | 3/15/20 | 5/11/20 | 3/14/20 |  | 3/15/20 |  | 3/15/20 | 4/13/20 |
| **Sweden** |  |  |  |  |  |  |  | 3/11/20 |  |  |  |
| **Switzerland** |  |  |  |  |  | 3/13/20 |  | 2/28/20 |  | 3/16/20 | 4/27/20 |
| **Turkey** |  |  |  |  |  | 3/16/20 |  | 3/16/20 |  |  |  |
| **UK** |  |  |  | 3/23/20 |  | 3/23/20 |  | 3/23/20 |  | 3/24/20 |  |
| **Ukraine** |  |  |  |  |  | 3/12/20 |  | 3/12/20 |  | 3/17/20 | 5/11/20 |
| **USA** |  |  |  |  |  |  |  |  |  |  |  |
|  | Alabama |  |  | 4/04/20 | 4/30/20 | 3/19/20 |  | 3/19/20 |  | 3/28/20 | 4/30/20 |
|  | Alaska | 3/28/20 |  | 3/28/20 | 4/24/20 | 3/16/20 |  | 3/24/20 | 5/22/20 | 3/28/20 | 4/24/20 |
|  | Arizona |  |  | 3/30/20 | 5/16/20 | 3/16/20 |  | 3/30/20 | 5/16/20 |  |  |
|  | Arkansas |  |  |  |  | 3/17/20 |  | 3/27/20 |  |  |  |
|  | California |  |  | 3/19/20 |  | 3/19/20 |  | 3/11/20 |  | 3/19/20 |  |
|  | Colorado |  |  | 3/26/20 | 5/09/20 | 3/23/20 |  | 3/19/20 |  | 3/26/20 | 5/09/20 |
|  | Connecticut |  |  |  |  | 3/17/20 |  | 3/12/20 |  | 3/23/20 | 5/20/20 |
|  | Delaware |  |  | 3/24/20 | 6/01/20 | 3/16/20 |  | 3/16/20 |  | 3/24/20 | 5/08/20 |
|  | District of Columbia |  |  | 3/30/20 | 5/29/20 | 3/16/20 |  | 3/13/20 |  | 3/25/20 | 5/29/20 |
|  | Florida |  |  | 4/03/20 | 5/18/20 | 3/17/20 |  | 4/03/20 | 6/05/20 |  |  |
|  | Georgia |  |  | 4/03/20 |  | 3/18/20 |  | 3/24/20 |  |  |  |
|  | Hawaii |  |  | 3/25/20 |  | 3/19/20 |  | 3/17/20 |  | 3/25/20 | 5/01/20 |
|  | Idaho |  |  | 3/25/20 | 5/01/20 | 3/23/20 |  | 3/25/20 | 5/01/20 | 3/25/20 | 5/01/20 |
|  | Illinois |  |  | 3/21/20 |  | 3/17/20 |  | 3/13/20 |  | 3/21/20 | 5/01/20 |
|  | Indiana |  |  | 3/25/20 | 5/18/20 | 3/19/20 |  | 3/12/20 |  | 3/24/20 | 5/18/20 |
|  | Iowa |  |  |  |  | 4/04/20 |  | 3/17/20 | 6/12/20 | 3/17/20 | 5/08/20 |
|  | Kansas |  |  | 3/30/20 | 5/04/20 | 3/17/20 |  | 3/17/20 |  |  |  |
|  | Kentucky |  |  |  |  | 3/20/20 |  | 3/19/20 |  | 3/26/20 | 5/11/20 |
|  | Louisiana |  |  | 3/23/20 | 5/15/20 | 3/16/20 |  | 3/13/20 | 5/15/20 | 3/22/20 | 5/01/20 |
|  | Maine |  |  | 4/02/20 | 5/31/20 | 3/16/20 |  | 3/18/20 |  | 3/25/20 | 5/01/20 |
|  | Maryland |  |  | 3/30/20 |  | 3/16/20 |  | 3/16/20 | 6/10/20 | 3/23/20 | 5/15/20 |
|  | Massachusetts |  |  |  |  | 3/17/20 |  | 3/13/20 |  | 3/24/20 | 5/18/20 |
|  | Michigan |  |  | 3/24/20 | 6/01/20 | 3/16/20 |  | 3/13/20 |  | 3/23/20 | 5/07/20 |
|  | Minnesota |  |  | 3/28/20 | 5/18/20 | 3/18/20 |  | 3/28/20 |  |  |  |
|  | Mississippi |  |  | 4/03/20 | 4/27/20 | 3/19/20 |  | 3/24/20 |  | 4/03/20 | 4/27/20 |
|  | Missouri |  |  | 4/06/20 | 5/15/20 | 3/23/20 |  | 3/23/20 |  |  |  |
|  | Montana |  |  | 3/26/20 | 4/26/20 | 3/15/20 |  | 3/24/20 | 6/01/20 | 3/26/20 | 5/01/20 |
|  | Nebraska |  |  |  |  | 4/02/20 |  | 3/16/20 |  |  |  |
|  | Nevada |  |  | 3/31/20 | 5/09/20 | 3/16/20 |  | 3/24/20 |  | 3/21/20 | 5/09/20 |
|  | New Hampshire |  |  | 3/27/20 |  | 3/16/20 |  | 3/16/20 |  | 3/28/20 | 5/11/20 |
|  | New Jersey |  |  | 3/21/20 | 6/09/20 | 3/18/20 |  | 3/16/20 |  | 3/21/20 | 5/02/20 |
|  | New Mexico |  |  |  |  | 3/13/20 |  | 3/12/20 |  | 3/24/20 | 5/15/20 |
|  | New York |  |  | 3/22/20 |  | 3/18/20 |  | 3/12/20 |  | 3/22/20 | 6/08/20 |
|  | North Carolina |  |  | 3/30/20 | 5/08/20 | 3/14/20 |  | 3/14/20 |  | 3/30/20 | 5/08/20 |
|  | North Dakota |  |  |  |  | 3/16/20 |  |  |  |  |  |
|  | Ohio |  |  | 3/23/20 | 5/20/20 | 3/16/20 |  | 3/12/20 |  | 3/23/20 | 5/04/20 |
|  | Oklahoma |  |  |  |  | 3/17/20 |  | 3/24/20 | 5/24/20 | 4/01/20 | 4/24/20 |
|  | Oregon |  |  | 3/23/20 |  | 3/16/20 |  | 3/12/20 |  |  |  |
|  | Pennsylvania |  |  | 4/01/20 | 6/05/20 | 3/17/20 |  | 4/01/20 |  | 3/23/20 | 5/08/20 |
|  | Puerto Rico |  |  | 3/16/20 |  | 3/16/20 |  | 3/12/20 |  | 3/16/20 | 5/04/20 |
|  | Rhode Island |  |  | 3/28/20 | 5/09/20 | 3/16/20 |  | 3/17/20 |  |  |  |
|  | South Carolina |  |  | 4/07/20 | 5/04/20 | 3/16/20 |  | 3/18/20 |  |  |  |
|  | South Dakota |  |  |  |  | 3/16/20 |  | 4/06/20 | 4/28/20 |  |  |
|  | Tennessee |  |  | 4/02/20 |  | 3/20/20 |  | 3/23/20 |  | 4/01/20 |  |
|  | Texas |  |  | 4/02/20 | 5/01/20 | 3/19/20 |  | 3/21/20 |  |  |  |
|  | Utah |  |  |  |  | 3/16/20 |  | 3/19/20 |  |  |  |
|  | Vermont |  |  | 3/24/20 | 5/15/20 | 3/18/20 |  | 3/13/20 |  | 3/25/20 | 5/04/20 |
|  | Virginia |  |  | 3/30/20 | 6/05/20 | 3/16/20 |  | 3/15/20 |  | 3/24/20 | 5/15/20 |
|  | Washington |  |  | 3/23/20 |  | 3/13/20 |  | 3/11/20 |  | 3/25/20 |  |
|  | West Virginia |  |  | 3/25/20 | 5/04/20 | 3/14/20 |  | 3/24/20 |  | 3/24/20 | 5/04/20 |
|  | Wisconsin |  |  | 3/25/20 | 5/13/20 | 3/18/20 |  | 3/17/20 |  | 3/25/20 | 5/11/20 |
|  | Wyoming |  |  |  |  | 3/19/20 |  | 3/20/20 |  |  |  |

UK:United Kingdom; USA: United States of America.
