# Supplementary figures and images for: COVID-19 pandemic and Farr’s law: A global comparison and prediction of outbreak acceleration and deceleration rates
Source: PLoS One. 2020 Sep 17;15(9):e0239175. doi: 10.1371/journal.pone.0239175 (PMC7498003; doi:10.1371/journal.pone.0239175)

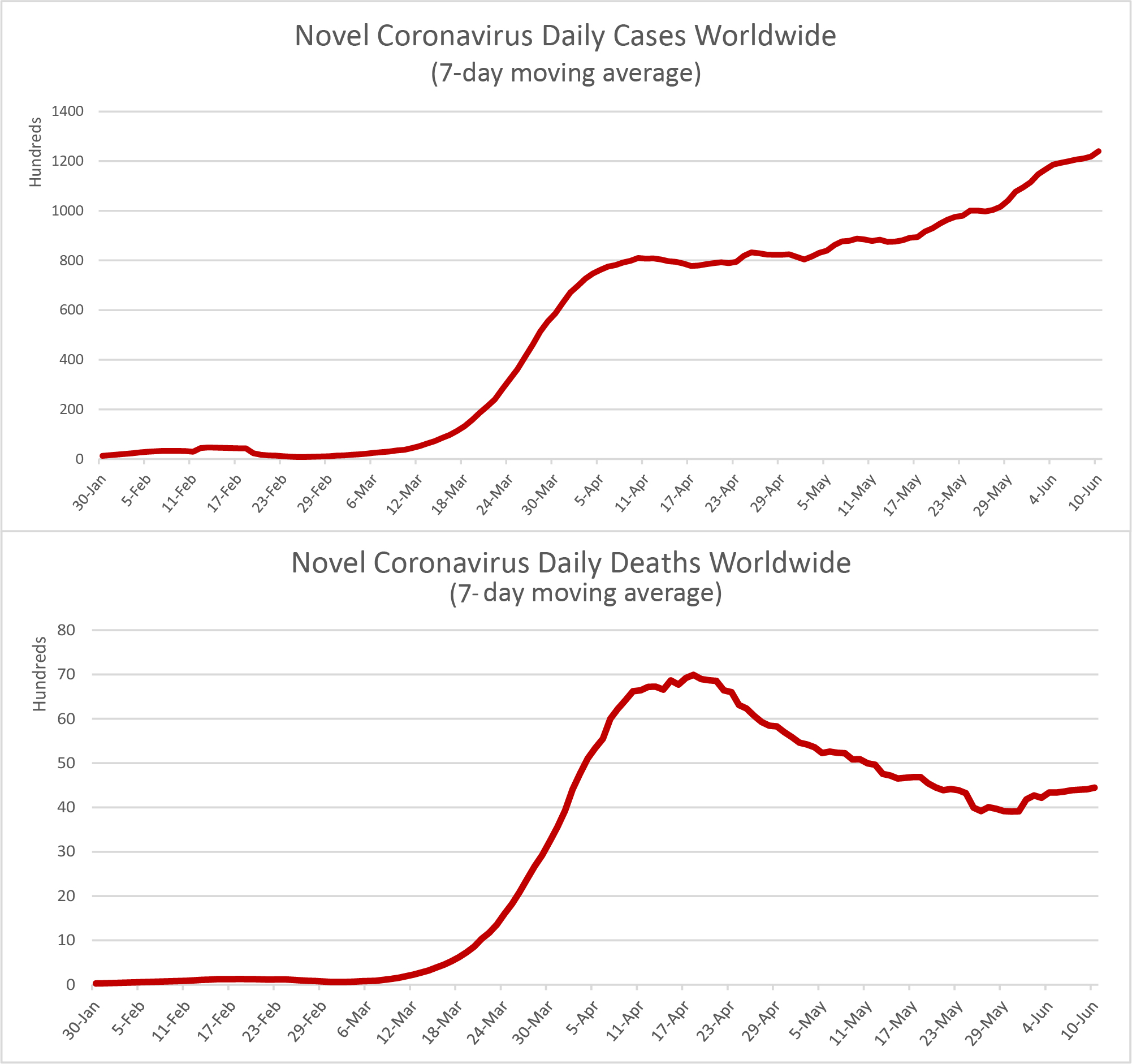

Supplement: S1 Fig — (A) New daily cases and (B) new daily deaths incidence. (TIF) [file pone.0239175.s005.tif]
