# Supplementary material for: COVID-19 pandemic and Farr’s law: A global comparison and prediction of outbreak acceleration and deceleration rates
Source: PLoS One. 2020 Sep 17;15(9):e0239175. doi: 10.1371/journal.pone.0239175 (PMC7498003; doi:10.1371/journal.pone.0239175)
Supplement: Attachment — Submitted filename: COVID-19 Pandemic and Farrs Law - final review.docx [file pone.0239175.s006.docx]

Reviewer: Dr. Farshad Farzadfar

Fregni et al. provided a study to investigate Farr’s law in the COVID-19 pandemic. The authors tried to come up with an efficient model to predict acceleration and deceleration rates of the COVID-19 outbreak and probable time of outbreak relative resolution.

Although the authors inspected mentioned notion well, several issues are necessary to be considered, including:

1. In the abstract, it is mentioned that COVID-19 data until April 10, 2020, is utilized for modeling, wasn’t is possible to use newer data for this study?
2. In the introduction, the authors mention that the importance of this study is to prepare the medical system against epidemics. Still, the final results of the study may implicate deceleration and endpoints of the epidemic more prominent. The main goal of the study should be highlighted.
3. Telling the life story of Mr. Farr in the introduction seems to be unnecessary for understanding the aim of this study. Besides, introducing components of Farr’s law in the introduction instead of the methods part can be more useful.
4. The first and second ratios (R_1_ and R_2_) are explained vaguely in the methods.
5. Are Worldometer website data on COVID-19 statistics reliable enough to use in such a study? Is there any previous evidence one quality of their statistics? Data quality? Different criteria for diagnosis?
6. Does the “predefined hypothesis that higher R1 and R2 ratios are correlated with higher numbers of cases or deaths” sentence has a reference? Especially about predicting deaths this claim is more questionable.
7. Data presented in S2 and S3 tablets are not consistent with prediction numbers for new cases and new deaths beyond 10^th^ June 2020.
8. The results of the study are not well discussed in the discussion part. More detailed benefits of the results of the study could help readers more, and offer more solutions for health policymakers.
9. Numbers of countries reaching values near zero in new cases and deaths are not discussed that make how much population and talking socioeconomic status of these countries could help more.
10. The conclusion part does not conclude the main message of the study. A warning message for high-risk areas, according to predictions discussed, could be more beneficial.
